# Integration of multi-omics data to elucidate keystone unknown taxa within microbialite-forming ecosystems

**DOI:** 10.3389/fmicb.2023.1174685

**Published:** 2023-07-28

**Authors:** Rocío Amorín de Hegedüs, Ana Conesa, Jamie S. Foster

**Affiliations:** ^1^Genetics Institute, University of Florida, Gainesville, FL, United States; ^2^Department of Microbiology and Cell Sciences, Space Life Sciences Lab, University of Florida, Merritt Island, FL, United States; ^3^Spanish National Research Council, Institute for Integrative Systems Biology, Valencia, Spain

**Keywords:** microbial dark matter, microbialites, metagenomics, metatranscriptomics, amplicon, networks

## Abstract

Microbes continually shape Earth’s biochemical and physical landscapes by inhabiting diverse metabolic niches. Despite the important role microbes play in ecosystem functioning, most microbial species remain unknown highlighting a gap in our understanding of structured complex ecosystems. To elucidate the relevance of these unknown taxa, often referred to as “microbial dark matter,” the integration of multiple high throughput sequencing technologies was used to evaluate the co-occurrence and connectivity of all microbes within the community. Since there are no standard methodologies for multi-omics integration of microbiome data, we evaluated the abundance of “microbial dark matter” in microbialite-forming communities using different types meta-omic datasets: amplicon, metagenomic, and metatranscriptomic sequencing previously generated for this ecosystem. Our goal was to compare the community structure and abundances of unknown taxa within the different data types rather than to perform a functional characterization of the data. Metagenomic and metatranscriptomic data were input into SortMeRNA to extract 16S rRNA gene reads. The output, as well as amplicon sequences, were processed through QIIME2 for taxonomy analysis. The R package mdmnets was utilized to build co-occurrence networks. Most hubs presented unknown classifications, even at the phyla level. Comparisons of the highest scoring hubs of each data type using sequence similarity networks allowed the identification of the most relevant hubs within the microbialite-forming communities. This work highlights the importance of unknown taxa in community structure and proposes that ecosystem network construction can be used on several types of data to identify keystone taxa and their potential function within microbial ecosystems.

## 1. Introduction

Often regarded as one of the planet’s first ecosystems, microbialites are organo-sedimentary structures formed as a result of trapping and binding activities of benthic microbial mat communities (Reid et al., 2000; Dupraz et al., 2009; Suarez-Gonzalez et al., 2019). Microbialites represent an important interface between the biosphere and geosphere, and mat communities that form these structures have been known to play an important role in planetary evolution by regulating global cycles of major elements, such as carbon, oxygen, nitrogen, and sulfur (Grotzinger and Knoll, 1999). There are several key functional groups of microbes within microbialite communities including oxygenic and anoxygenic phototrophs, aerobic heterotrophs, sulfate reducers, methanogens, and fermenters (Dupraz and Visscher, 2005; Baumgartner et al., 2009; Dupraz et al., 2009; Myshrall et al., 2010; Babilonia et al., 2018). However, studies on microbialite-forming microbial communities have shown that ~30% of the taxa recovered from high-throughput sequencing efforts are unclassified at the phyla level and more than 60% of the recovered genes and transcripts are of unknown function (Louyakis et al., 2018) demonstrating the need for improved approaches to explore these unknown taxa.

Colloquially named “microbial dark matter,” these unknown elements of microbial life drastically limit our understanding of microbial life and diversity, as well as the metabolic inner workings of microbial-dominated ecosystems, such as microbialite communities (Marcy et al., 2007; Rinke et al., 2013; Lok, 2015). Since much of our microbial knowledge is derived from relatively few cultivable taxa, this has resulted in a biased and limited view of the genetic and metabolic capabilities of microbial life. However, advances in sequencing technologies have been instrumental in studying these unknown and uncultured microbes, allowing us to sequence microorganisms eluding cultivation. The most common methods to study microbial communities include amplicon, metagenomic and metatranscriptomic sequencing. Each methodology is chosen according to the scientific questions and the needs of each individual study leading to the discovery of new genes, metabolic pathways, and taxa (Schulz et al., 2017; Bernard et al., 2018; Jiao et al., 2021). For instance, metagenomic sequencing has yielded evidence of dozens of new phyla as well as thousands of new taxa (Brown et al., 2015; Hug et al., 2016). Despite this progress, it is unclear how these methods can be integrated to better understand the role of unknown taxa, as meta-omic datasets are large, noisy, and not easy to mine or interpret (Marx, 2013; Parks et al., 2017).

To analyze ecosystem structure, microbial communities can be modeled as networks, where taxa are represented by nodes and their relationships are represented as edges, respectively. Networks effectively capture the general structure of the ecosystem, and provide information of the importance of different taxa within the community (Proulx et al., 2005). The intricate relationship between microorganisms within a microbial community can be analyzed using different network metrics that represent co-occurrence (degree centrality), connectivity (betweenness centrality) and centrality (closeness centrality), thus providing a measurement of how taxa within the ecosystem operate (Proulx et al., 2005; Ma'ayan, 2011; Ma et al., 2016). Keystone species that have high levels of all three of these metrics are “hubs” that contribute to maintain the network structure. Since their removal impacts the network connectivity, they are believed to hold ecological relevance within the ecosystem (Berry and Widder, 2014). In a previous study we developed MDMnets, a methodology that utilizes network theory to model microbial communities from amplicon data leading to the identification of unknown hub species that play a central role in the microbial communities (Zamkovaya et al., 2021).

In this study, we tested the pipeline MDMnets on three different types of data (amplicon, metagenome and metatranscriptome) from diverse microbialite-forming communities across the globe to investigate the contribution of “microbial dark matter” within these communities. Networks were analyzed for every combination of dataset and taxonomic classification level, both including and excluding the microbial dark matter component. For every dataset, a hub score was calculated for every node. Top scoring hubs were evaluated based on taxonomy and compared across the different methods. By identifying those currently unknown taxa that are forming potentially synergistic connections within the communities, we can prioritize important taxa for characterization, allowing us to improve our understanding of complex microbial ecosystems.

## 2. Materials and methods

### 2.1. Data obtention

Previously generated amplicon, metagenome and metatranscriptome data sets from microbialite-forming communities across the globe were selected for this study and retrieved from NCBI through the use of the NCBI SRA Toolkit. These consisted of 14 amplicon samples derived from thrombolites of Highborne Cay, The Bahamas (Mobberley et al., 2015) and microbialite samples from Storrs Lake, The Bahamas (Paul et al., 2016), where the V1-V3 region of the 16S rRNA gene was targeted for sequencing with 454 GS FLX pyrosequencing (NCBI accession numbers PRJNA305634 and PRJNA222307, respectively). In addition, 55 stromatolite metagenomic samples derived from locations throughout the Exuma Cays in The Bahamas, including Highborne Cay, Little Darby Island, Lee Stocking Island, and Bock Cay and from Hamelin Pool, Western Australia were used (Casaburi et al., 2016; Babilonia et al., 2018; NCBI accession numbers SRS1019263). Finally, 24 metatranscriptomic samples from Highborne Cay thrombolites generated over the diel cycle in three different seasons were also used (Louyakis et al., 2018; NCBI accession number PRJNA305634).

### 2.2. Data processing

#### 2.2.1. Amplicon sequencing analysis

Reads from the amplicon dataset (*n* = 14) were first run through quality control using cutadapt (v3.4) with options-q *30-u 20-m 50*. The resulting reads were processed through software QIIME2 (v2022.8; Bolyen et al., 2019). Amplicon reads were denoised using dada2 *denoise-pyro* algorithm plugin on the QIIME2 platform, which works by denoising, de-replicating and filtering chimeras from sequences generated by pyrosequencers (Callahan et al., 2016). Next, taxonomy assignment was performed utilizing the *feature-classifier sklearn* plugin in QIIME2, which works by training a Naive Bayes classifier using reference sequences, in this case the SILVA reference sequences (www.arb-silva.de, v138; Quast et al., 2013). The resulting taxa abundance matrix was filtered to retain those OTUs present in at least two different samples. In addition, taxa classified as Eukaryota at the domain level, or as a chloroplast at the order to genus levels, were removed from the analysis.

#### 2.2.2. Metagenomic and metatranscriptomic sequencing analysis

Quality control of raw reads was performed with software FastQC (v0.11.7; Andrews, 2014) and posterior trimming/filtering of low-quality reads was done with cutadapt (v3.4; Martin, 2011). The metagenomic and metatranscriptomic datasets were processed with SortMeRNA (v4.3.6) to extract the 16S and 18S rRNA genes with default parameters against the smr_v4.3_default_db.fasta (Kopylova et al., 2012). The obtained reads were assembled using Spades (v3.15.3) with a size cutoff of 220 nt and analyzed with QIIME2 (v2022.8; Bolyen et al., 2019). The resulting 16S rRNA gene reads were dereplicated using *vsearch* plugin dereplicate-sequence. In this step, identical sequences were grouped to obtain a non-redundant set of sequences. Next, features were clustered into OTUs with a percent threshold identity of 99% using the SILVA SSU reference database (www.arb-silva.de, v138.1; Quast et al., 2013). In order to ensure that informative reads were used for taxonomic classification, reads were filtered utilizing *qiime quality-control exclude-seq* plugin using *vsearch* as a method, the SILVA SSU reference database as a reference, a percent identity threshold of 90% and a percent of query aligned threshold of 90%. Posterior taxonomy assignment was done utilizing the *feature-classifier classify-consensus-vsearch* plugin in QIIME2. Next, the reads were mapped back to the contigs using bowtie2(v2.4.5) to estimate abundance. Taxa classified as Eukaryota at the domain level were filtered out from the study for a better comparison of the known taxa. In addition, taxa classified as chloroplasts at the order, family or genus levels were removed from the analysis. Finally, taxa (i.e., known and unknown) that were present in at least 15 different samples (62.5% of the total) for metatranscriptome data and 34 different samples (61.8% of the total) for metagenome data were considered for the downstream analysis.

#### 2.2.3. Identification of microbial dark matter

Specific keywords were searched for within the taxonomic classifications to classify taxa as “microbial dark matter” and make the labels uniform across the datasets. Classifications containing the words “uncultured,” “unknown,” “NA,” “unknown_family,” “Incertae Sedis,” amongst others, were re-classified as “microbial dark matter” at the taxonomic level at which this classification occurred and all the subsequent levels. For example, a given taxa classified as “unknown” at the order level was re-classified as “microbial dark matter” at the order, family, genus, and species levels.

### 2.3. Network construction, edge evaluation and filtering

All subsequent statistical and network analyses were conducted in R (v 4.2.2) using packages Phyloseq (v1.42.0; McMurdie and Holmes, 2013), qiime2R (v0.99.6; Bisanz, 2018), dada2 (v.1.26.0; Callahan et al., 2016), SpiecEasi (v1.1.2; Kurtz et al., 2015), igraph (v1.3.5; Csardi and Nepusz, 2006), and mdmnets (v0.1.0; Zamkovaya et al., 2021). Networks were constructed using R package mdmnets, which normalizes the abundance tables obtained from QIIME2 and converts them into an adjacency matrix using R package SpiecEasi. Afterwards, Meinshausen-Buhlmann’s neighborhood selection method (Kurtz et al., 2015) was utilized to calculate conditional dependence between each OTU pair. Edges represent linear relationships between two nodes that are not conditionally independent. This method is recommended for microbiome data, as it is designed for sparse and compositional data and reduces the probability of spurious relationships (Kurtz et al., 2015). Network edge significance for all three dataset types was evaluated using a bootstrap approach. First, the original networks were constructed using the OTU abundance tables obtained from QIIME2. To build the bootstrap networks, the abundances for each OTU were randomly sampled to break the associations between sample and abundance. This process was repeated 5,000 times, and the number of times each edge from the original network appeared in the bootstrap networks was noted. Edges appearing in more than 250 bootstrap networks (*p* ≥ 0.05) were filtered out from the original network to obtain a filtered network. Next, hub score metrics were extracted from the filtered network using the SpiecEasi hub_score() function to identify taxa that are playing significant roles in network structure. The resulting matrices were visualized as networks using functions from R package SpiecEasi with nodes representing OTUs and edges representing direct co-occurrence relationships between them.

### 2.4. Network analysis and hub score evaluations

For each dataset, unknowns were removed from the OTU tables to generate plots without “microbial dark matter” for comparison purposes. Additionally, bootstrap networks were generated through the use of R package mdmnets function comp_by_deleting_random_knowns_t_v3() to confirm metric changes are caused by removing unknown taxa and not by changes in the network size (Zamkovaya et al., 2021). This function generated a total of 100 bootstrap networks through the removal of random known taxa and calculated the network metrics in all the generated bootstrap networks. The statistical significance of the changes across networks was tested using a Wilcox test. This analysis was performed at each taxonomic level from phylum to genus.

For each dataset, hub scores were extracted for each node composing the network. The list of top 20 scoring hubs from each dataset, with and without unknown taxa, was taxonomically annotated and compared across data modalities. The unknown taxa present within the top 20 scoring hubs for each dataset was analyzed through BLAST to evaluate similarities to other taxa present in the database.

## 3. Results

### 3.1. Sample and data processing

The amplicon dataset had on average ~ 22,000 reads per sample and after trimming and filtering, 4.21% were removed from downstream analyses (Supplementary Table 1). The metagenomic samples had an average of ~24,000,000 reads per sample and after processing with Cutadapt, 90.71% of the reads were retained and utilized for 16S/18S rRNA gene sorting. A total of 267,392 reads (1.17%) of these reads were identified as small subunit rRNA genes (SSU) and used as input for QIIME2 (Supplementary Table 1). Finally, the metatranscriptomic dataset contained an average of ~25,000,000 reads per sample and quality control resulted in 94.65% of these reads being retained for subsequent analyses. For the metatranscriptomic dataset, 66.5% of the filtered reads were recognized by SortMeRNA as SSU reads due to the high expression levels of the rRNA genes within the communities (Supplementary Table 1).

### 3.2. Taxonomic characterization of thrombolites using different-omics approaches revealed different levels of unknown taxa at each classification level

**Figure 1:**
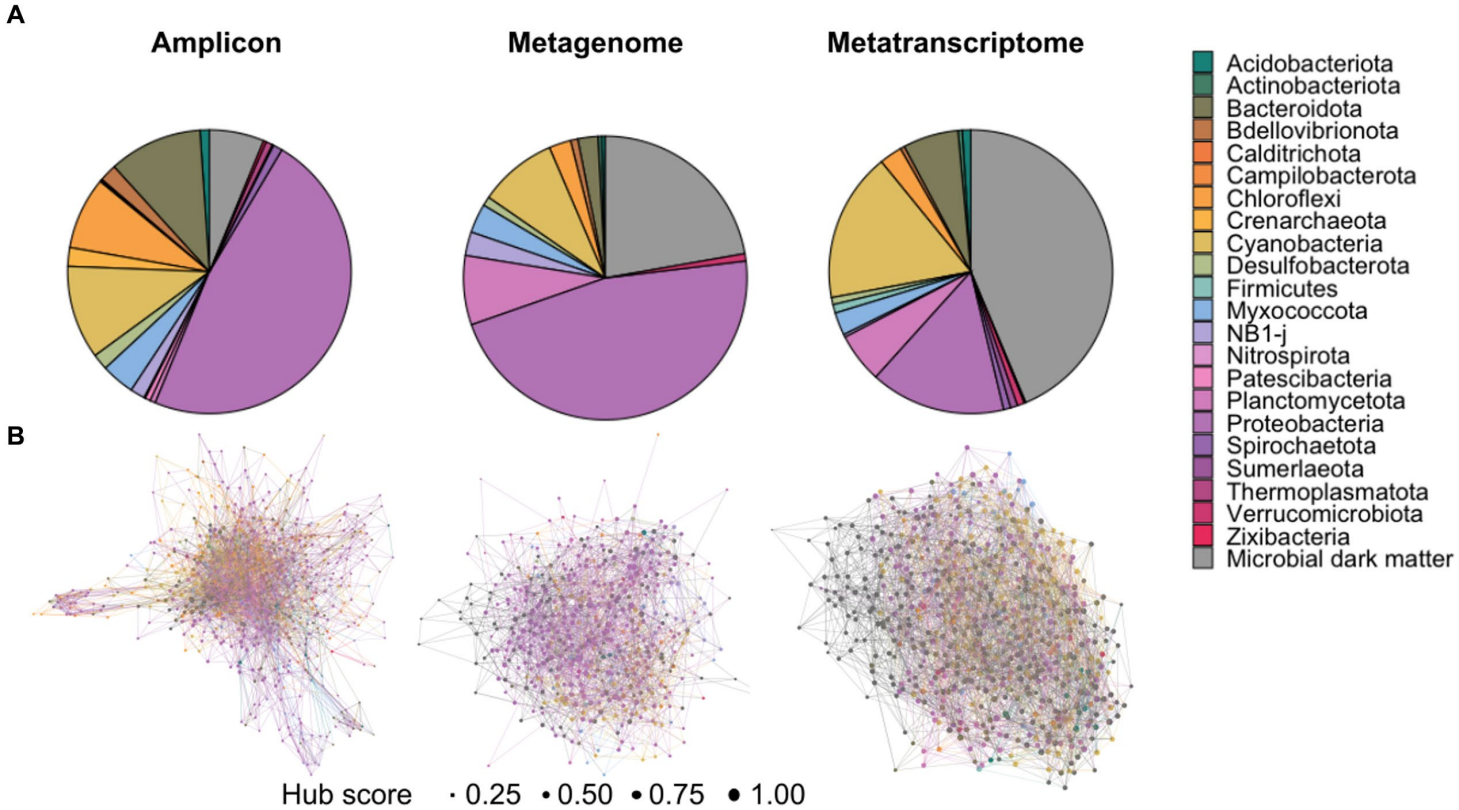
Overall diversity within the amplicon, metagenomic and transcriptomic datasets. **(A)** Distribution of phyla abundance in each dataset demonstrating a high proportion of “microbial dark matter” present in the metagenomic and metatranscriptomic datasets. **(B)** Hub network at the phyla level, hub size indicates hub score, with one being highest hub score and zero being lowest. Each network represents the three types of data depicting the distinctive patterns of unknown organisms, labeled as “microbial dark matter,” within the microbialite communities.

Across all data types, analyses revealed that unknown classifications were more prevalent at each successive classification level (Figure 1; Supplementary Figures 1–4). Please note that although there have been recent updates to the nomenclature of several phyla of prokaryotes (Oren and Garrity, 2021), the terms listed in the text and figures reflect the current annotations in the SILVA database. In the amplicon datasets, a total of 680 taxa were present in at least two samples. At the domain level, most of the OTUs were identifiable as Bacteria (93.8%), followed by Archaea (2.7%) and Eukaryota (0.14%), with 3.2% of the community unable to be assigned to a domain. At the phyla level, most of these taxa were assigned to a known classification, most frequently Proteobacteria (i.e., Pseudomonadota) and Cyanobacteria, with only 6.25% assigned as unknowns (Figure 1A). At the genus level, however, only 195 (28.6%) of these taxa were assigned to a known organism, with only 16 (2.3%) being annotated at the species level (Supplemental Figure 4).

Within the metagenomic datasets we found 506 distinct taxa present in at least 34 of the samples. The majority of these taxa were classified as bacteria at the domain level (75.88%). At least 35.5% of them were classified as Bacteria at this level, and 20.5% were unassigned at domain level with none being classified as Archaea. The phyla level, however, showed that Proteobacteria was the most abundant taxa with 44.26%, followed by “unknown” with 21.14% and Cyanobacteria with 9.88% (Figure 1A). Only 11 (2.17%) of these taxa were classified at the species level.

For the metatranscriptomic dataset, 1,383 different taxa were found in at least 15 of the samples. Unlike the previous datasets, however, a large majority of the taxa from the metatranscriptomic datasets were classified as Eukaryota (52%), followed by Bacteria (28.41%) and the rest were unassigned (19.45%). Accordingly, the most abundant classification at the phylum level was unknown organisms (23.93%), followed by Cyanobacteria (10.91%), and Proteobacteria (6.86%) amongst others (Figure 1A). Very few taxa (6.86%) were classified to the species level within the metatranscriptomic datasets. A table with the full taxonomic classification for all datasets is listed in Supplementary Dataset 1.

### 3.3. Comparison between all three methodologies revealed shared taxa between datasets

**Figure 2:**
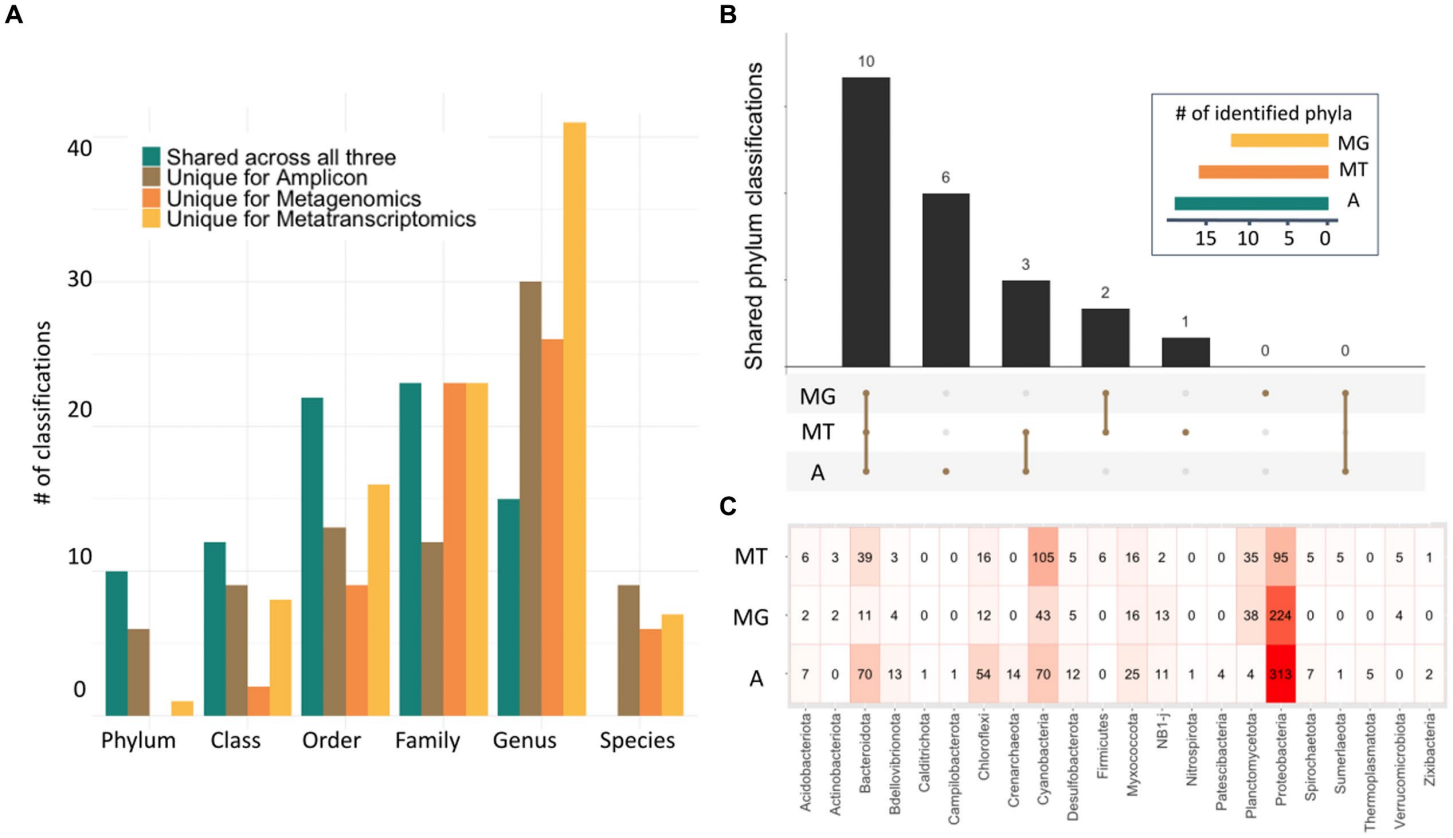
Characterization of microbialite composition with three-omics sequencing technologies. **(A)** Bar plot depicting unique taxonomy classifications found in each dataset for each taxonomic level, as well as those shared across all three methods. **(B)** Upset plot representing the number of common classifications found for the amplicon (A), metagenomic (MG) and metatranscriptomic (MT) data in the phylum rank (inset). **(C)** Heatmap at the phylum level showing the differences in identification and abundances in all three classifications with those phyla with high abundances in pink and red.

Although these datasets originated from different microbialite communities from across the globe, several shared taxa between all microbialite datasets were found (Figures 2A,B). For example, after removing taxa classified as Eukaryota, the number of identified taxa was 679, 481, and 616 for amplicon, metagenomic and metatranscriptomic, respectively. A comparison of the different phyla found in all three datasets showed that most phyla were identified by all three methods (Figure 2A). Additionally, the 10 phyla identified by all technologies (Proteobacteria, Bacteroidota, Myxococcota, Cyanobacteria, Planctomycetes, Chloroflexi, Desulfobacterota, NB1-j, Acidobacteriota and Bdellovibrionota) also correspond to the most abundant phyla in all three methods (Figure 2C). At least seven phyla were uniquely identified by one method, with six being identified only by Amplicon (Crenarchaeota, Thermoplasmatota, Nitrospirota, Campilobacterota, Calditrichota, Patescibacteria) and one being identified only by metatranscriptomics (Firmicutes). Lastly, five phyla were identified by only two methods, with Sumerlaeota, Spirochaetota and Zixibacteria being identified in the amplicon and metatranscriptomics datasets and Actinobacteriota and Verrucomicrobiota being identified by both metagenome and metatranscriptome data (Figure 2B). Additionally, this same comparison was done at the subsequent taxonomic classification levels showing that up to the family level, most taxa were identified by all three methods, with metatranscriptomics identifying more different genus classifications and amplicon identifying more species. Consistently, metagenomics appears to identify the least unique taxa across all classification levels (Figure 2A). Comparisons for each taxonomic level can be seen in Supplementary Figure 5.

### 3.4. Edge filtering through bootstrap analysis

R package mdmnets utilizes taxa abundance tables to construct networks by analyzing co-occurrence relationships between operational taxonomic units (OTU) through the SpiecEasi Meinshausen-Buhlmann neighborhood algorithm, which estimates conditional dependence between each pair of OTUs. This approach is ideal for sequencing data that is sparse and compositional, as it prevents spurious relationships from being included in the network. The pipeline outputs an adjacency matrix describing the relationships and can be represented as a network. Networks were constructed with and without unknown taxa, to investigate the importance of unknowns in network structure (Figure 1B). For the amplicon dataset, the initial network had 4,160 links out of which 3,548 (85.2%) were significant (*p* < 0.05), and the non-significant edges were removed from the final network. For both metagenomic (2,167 links) and metatranscriptomic (3,559 links) data all edges were significant (*p* < 0.05). The obtained networks for the phyla level can be seen in Figure 1B, whereas networks for other classification levels can be found in Supplementary Figures 1–4.

### 3.5. “Microbial dark matter” was critical for network integrity across all data types

**Figure 3:**
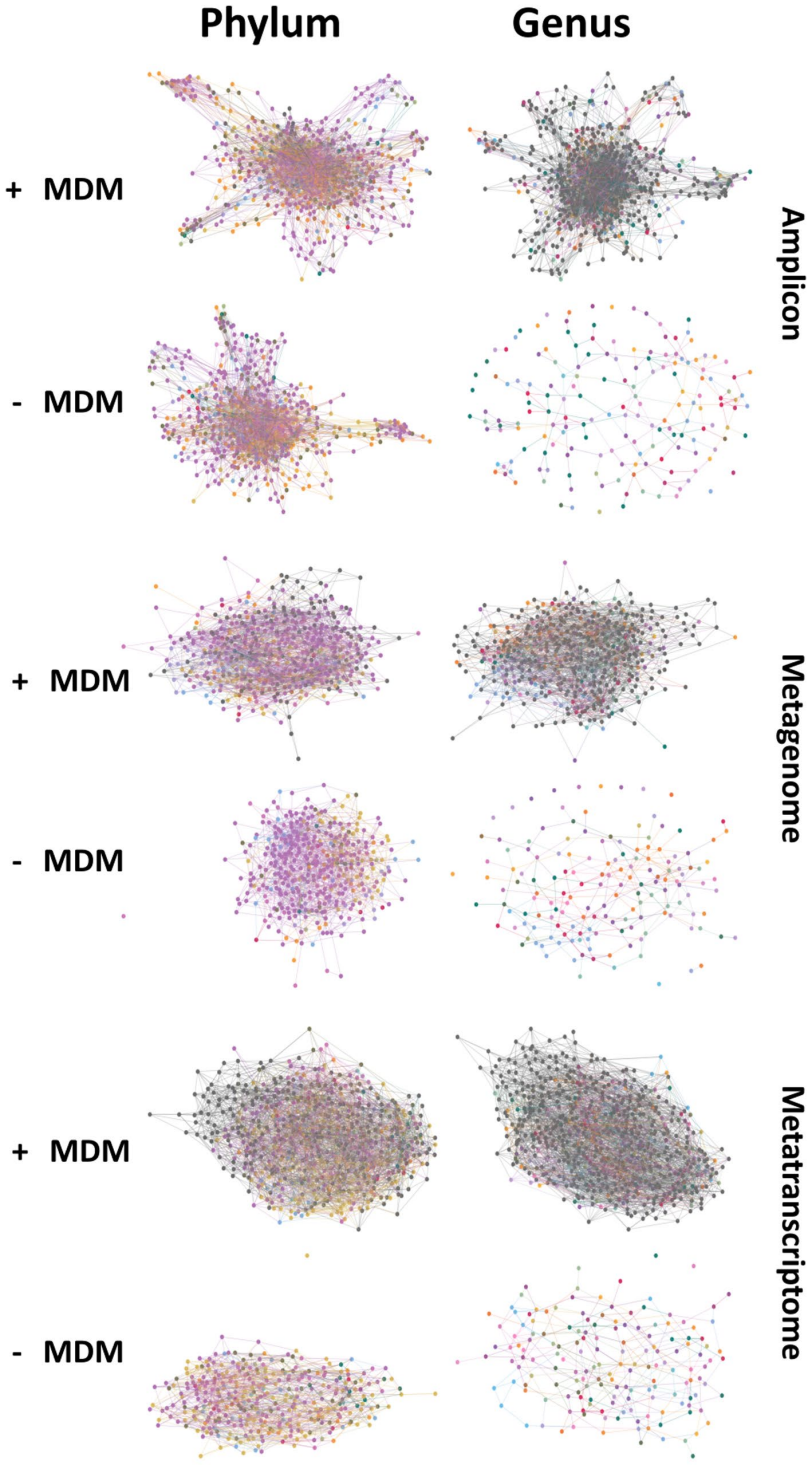
Networks created at the phyla and genus level for amplicon, metagenomic and metatranscriptomic datasets showing structural changes in network connectivity when comparing networks created with and without microbial dark matter (MDM).

Unknown taxa appeared to play a role in maintaining network structure at all taxonomic levels in all data types, with varying degrees of importance (Figure 3; Supplementary Figures 6–10). The removal of unknown taxa caused network fragmentation and disruption of the connectivity between nodes (Figure 3). For all three methods, fragmentation in absence of the unknown taxa appeared at higher taxonomic levels. At genus level all three datasets had extremely fragmented networks after unknown node removal, as is expected given the large number of unknown taxa at this level in comparison to other taxonomic levels Supplementary Figure 10.

**Figure 4:**
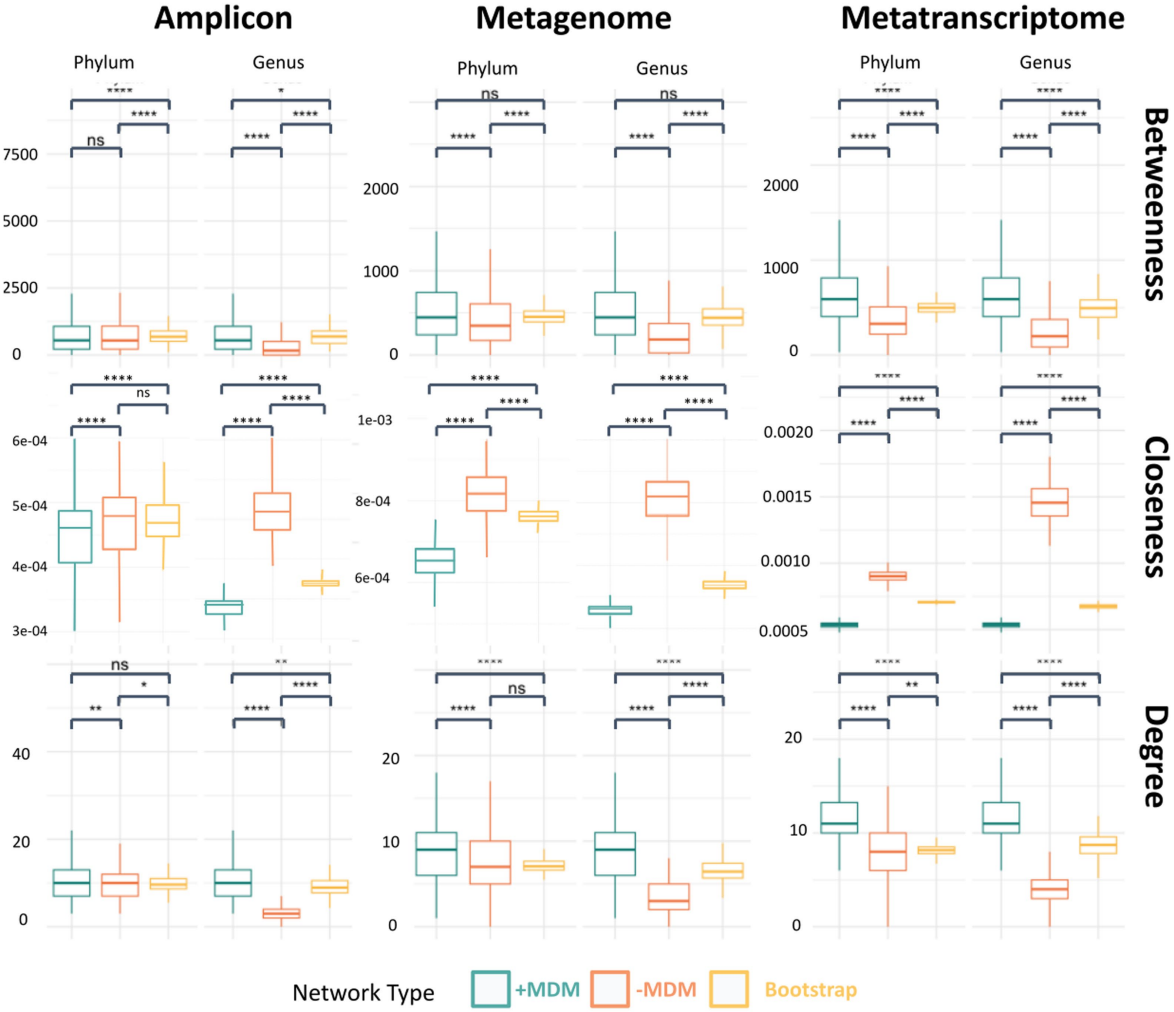
Comparison of centrality scores for the metatranscriptomic dataset between the original network, the network without unknown taxa and the bootstrap network at the phylum and genus levels. The evaluated network metrics of the hubs represent connectivity including betweenness centrality, closeness centrality and co-occurrence (i.e., degree centrality; ns, *p* > 0.05; *, *p* ≤ 0.05; ** *p* ≤ 0.01; ***, *p* ≤ 0.001; ****, *p* ≤ 0.0001).

These network changes can be evaluated through analyzing different network metrics, such as degree centrality, closeness centrality and betweenness centrality and comparing these values between the original networks and the networks without the unknown taxa. To evaluate if changes in network metrics were specifically due to the importance of unknowns within the community and not because of the removal of many nodes, a bootstrap network was created (Figure 4; Supplemental Figures 11–13). Network metrics in the original network, the network without the unknowns and the bootstrap networks are shown in Figure 4 for phylum and genus taxonomic levels. The constructed metatranscriptomic networks without unclassified taxa showed drastic changes in betweenness centrality, degree and closeness centrality when compared to the original and the bootstrap networks for all taxonomic levels. These differences were expected as the number of unknown taxa within the network was large enough that any similar number of OTUs removed affected the network structure. However, the centrality scores for the network without unknown taxa were lower than the bootstrap network suggesting that these taxa also hold relevance within the community, as the exclusion of random nodes from the network did not have the same effect as the unknown nodes being removed.

The metagenomic dataset showed similar results, with less drastic but still significant changes in centrality scores when compared to metatranscriptomic data, which was expected given that the number of unknown nodes was much lower in proportion. Amplicon networks, however, only showed similarities in the lower taxonomy levels when compared to original and bootstrap networks, showing larger differences in centrality scores when approaching higher taxonomy levels.

### 3.6. Evaluation of top scoring hubs revealed unknown taxa occupy important roles within ecosystem for all data types

**Figure 5:**
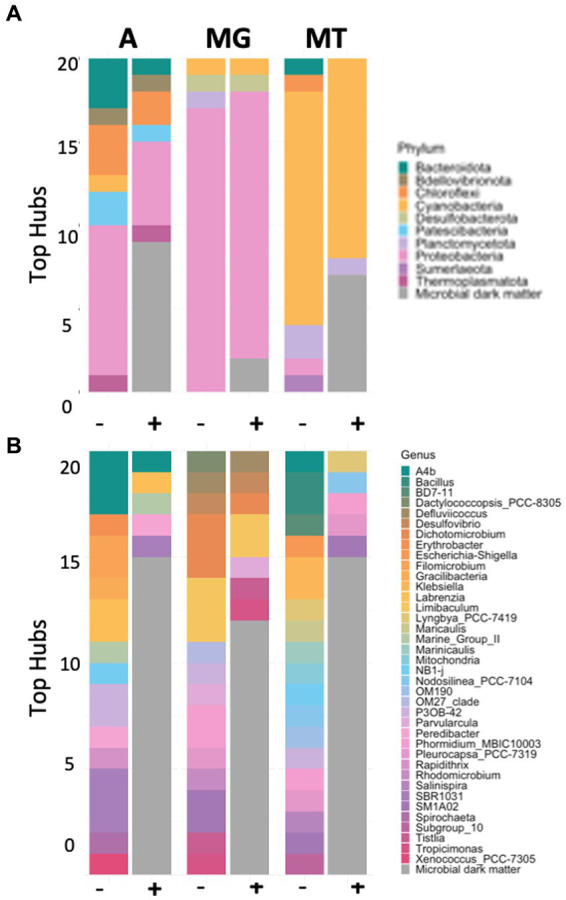
Bar plots showing microbial diversity in phylum **(A)** and genus **(B)** for top 20 hubs in the presence (+) and absence (-) of microbial dark matter in the amplicon (A), metagenomic (MG), and metatranscriptomic (MT) data sets.

Top scoring hubs were analyzed to evaluate the presence of known and unknown taxa amongst the most highly connected hubs within the community (Figure 5; Supplemental Figures 14–16). First, we focused on those hubs that corresponded to known taxa at the phylum, class, order, family, and genus levels. The known top scoring hubs for amplicon and metagenomic data were enriched in the phylum Proteobacteria (Figure 5). Furthermore, the amplicon and metatranscriptomics datasets contained the highest number of different phyla, while metagenomics contained only four different classifications. Furthermore, known metatranscriptomics hubs were enriched in Cyanobacteria, and included phyla Bacteroidota, Chloroflexi, Planctomycetota, Proteobacteria and Sumerlaeota. These taxa likely represent the most metabolically active phyla in this environment.

This pattern of increased diversity for the amplicon and metatranscriptomics datasets could be observed when looking at the top scoring known hubs for subsequent classification levels (Supplementary Figures 14–16). The inclusion of the unknown taxa into the analysis allowed us to analyze the prevalence of unknown organisms amongst the top scoring hubs (Table 1). For the amplicon data set, 40% of the top 20 scoring hubs were unclassified at the domain level and four of these unclassified taxa were in the top five, suggesting the top scoring hubs likely belong to previously undescribed lineages. The remaining taxa in the top 20 belonged mostly to the domain Bacteria, and phyla Proteobacteria, Chloroflexi and Bacteroidota. Moreover, one hub classified as Archaea phyla Thermoplasmatota was also found amongst the top 20 scoring hubs. The taxa that were unclassified at the domain or phyla level were blasted against the NCBI database to investigate similarities to other existing taxa. Only one hub had no significant matches, the rest matched with a similarity >95% to “uncultured bacterium,” “uncultured organism” or “uncultured delta proteobacterium,” indicating that, despite this taxon being unknown, they had been previously observed in other ecosystems.

**Table 1:** Classification for all taxonomic levels of the top 20 highest scoring hubs found in the amplicon dataset.

Score	Domain	Phylum	Class	Order	Family	Genus	Species
1	MDM	MDM	MDM	MDM	MDM	MDM	MDM
0.747	Bacteria	Chloroflexi	Anaerolineae	SBR1031	A4b	A4b	MDM
0.684	MDM	MDM	MDM	MDM	MDM	MDM	MDM
0.643	MDM	MDM	MDM	MDM	MDM	MDM	MDM
0.611	MDM	MDM	MDM	MDM	MDM	MDM	MDM
0.611	Bacteria	Bdellovibrionota	Bdellovibrionia	Bacteriovoracales	Bacteriovoracaceae	*Peredibacter*	MDM
0.556	MDM	MDM	MDM	MDM	MDM	MDM	MDM
0.540	MDM	MDM	MDM	MDM	MDM	MDM	MDM
0.532	Bacteria	Proteobacteria	Alphaproteobacteria	Rhodobacterales	Rhodobacteraceae	MDM	MDM
0.518	Bacteria	Proteobacteria	Alphaproteobacteria	MDM	MDM	MDM	MDM
0.517	Bacteria	Proteobacteria	Alphaproteobacteria	Rhodobacterales	Rhodobacteraceae	MDM	MDM
0.478	Bacteria	MDM	MDM	MDM	MDM	MDM	MDM
0.477	Bacteria	Bacteroidota	Bacteroidia	Cytophagales	Amoebophiliaceae	MDM	MDM
0.475	Bacteria	Proteobacteria	Alphaproteobacteria	Rhizobiales	Stappiaceae	*Labrenzia*	MDM
0.464	MDM	MDM	MDM	MDM	MDM	MDM	MDM
0.460	Bacteria	Proteobacteria	Alphaproteobacteria	Rhodospiralles	Magnetospiraceae	MDM	MDM
0.454	Bacteria	Patescibacteria	Gracilibacteria	MDM	MDM	MDM	MDM
0.452	Bacteria	Chloroflexi	Anaerolineae	SBR1031	SBR1031	SBR1031	MDM
0.447	MDM	MDM	MDM	MDM	MDM	MDM	MDM
0.445	Archaea	Thermoplasmota	Thermoplasmata	Marine Gp II	Marine Gp II	Marine Gp II	MDM

MDM, microbial dark matter, or unknown taxa.

In the metagenomic data known taxa dominated the higher scoring hubs at least at the phylum level, with none of them classified at the species level (Table 2). Despite this, two of the top scoring hubs were unclassified at the domain level, and four were unclassified at the order level. The classified hubs belonged mostly to the phyla Proteobacteria with two hubs belonging two phyla Desulfobacteria and Cyanobacteria. A BLAST search of the two taxa unclassified at the domain level resulted in most of them matching to different uncultured bacterium.

**Table 2:** Classification for all taxonomic levels of the top 20 highest scoring hubs found in the metagenomic dataset.

Score	Domain	Phylum	Class	Order	Family	Genus	Species
1	Bacteria	Proteobacteria	Alphaproteobacteria	MDM	MDM	MDM	MDM
0.974	Bacteria	Proteobacteria	Alphaproteobacteria	Rhodobacterales	Rhodobacteraceae	*Limibaculum*	MDM
0.782	Bacteria	Proteobacteria	Alphaproteobacteria	Thalassobaculales	MDM	MDM	*MDM*
0.759	Bacteria	Proteobacteria	Alphaproteobacteria	Kiloniellales	Kiloniellaceae	*Tistlia*	MDM
0.727	Bacteria	Proteobacteria	Alphaproteobacteria	Thalassobaculales	MDM	MDM	MDM
0.722	Bacteria	Desulfobacteria	Desulfovibrionia	Desulfovibrionales	Desulfovibrionaceae	*Desulfovibrio*	MDM
0.705	Bacteria	Cyanobacteria	Cyanobacteria	Cyanobacteriales	Xenococcaceae	MDM	MDM
0.686	Bacteria	Proteobacteria	Alphaproteobacteria	Rhizobiales	Rhizobiales_Incertae_Sedis	*Dichotomicrobium*	MDM
0.683	Bacteria	Proteobacteria	Alphaproteobacteria	Kiloniellales	Kiloniellaceae	MDM	MDM
0.611	MDM	MDM	MDM	MDM	MDM	MDM	MDM
0.588	Bacteria	Proteobacteria	Alphaproteobacteria	MDM	MDM	MDM	*MDM*
0.588	Bacteria	Proteobacteria	Alphaproteobacteria	MDM	MDM	MDM	MDM
0.544	Bacteria	Proteobacteria	Alphaproteobacteria	Rhodobacterales	Rhodobacteraceae	*Tropicimonas*	MDM
0.541	Bacteria	Proteobacteria	Alphaproteobacteria	Caulobacterales	Parvularculaceae	*Parvularcula*	MDM
0.540	Bacteria	Proteobacteria	Alphaproteobacteria	Tistrellales	Geminicoccaceae	MDM	MDM
0.524	Bacteria	Proteobacteria	Alphaproteobacteria	Rhodobacterales	Rhodobacteraceae	MDM	*MDM*
0.520	Bacteria	Proteobacteria	Alphaproteobacteria	Defluviicoccales	Defluviicoccaceae	*Defluviicoccus*	MDM
0.514	Bacteria	Proteobacteria	Alphaproteobacteria	Rhodobacterales	Rhodobacteraceae	*Limibaculum*	MDM
0.512	MDM	MDM	MDM	MDM	MDM	MDM	MDM
0.509	Bacteria	Proteobacteria	Alphaproteobacteria	MDM	MDM	MDM	*MDM*

MDM, microbial dark matter, or unknown taxa.

Expectedly, the metatranscriptomic data was different from the metagenomic and amplicon data. Seven of the top 20 scoring hubs (Table 3) were unclassified at the domain level, and five were unclassified at the order or family levels. The classified taxa belonged mostly to phyla cyanobacteria, with one of them belonging to phylum Planctomycetota. Most of the unknown hubs matched to sequences to different cyanobacterium, uncultured fungus, or uncultured bacterium sequences.

**Table 3:** Classification for all taxonomic levels of the top 20 highest scoring hubs found in the metatranscriptomic dataset.

Score	Domain	Phylum	Class	Order	Family	Genus	Species
1	Bacteria	Cyanobacteria	Cyanobacteria	Phormisdemiales	Nodosilineaceae	*Nodosilinea_PCC-7104*	MDM
0.925	Bacteria	Cyanobacteria	Cyanobacteria	Phormisdemiales	Phormidesmiaceae	*Phormidium_MBIC10003*	MDM
0.882	Bacteria	Cyanobacteria	Cyanobacteria	MDM	MDM	MDM	MDM
0.871	Bacteria	Cyanobacteria	Cyanobacteria	Cyanobacteriales	Xenococcaceae	*Pleurocapsa_PCC-7,319*	MDM
0.843	Bacteria	Cyanobacteria	Cyanobacteria	Cyanobacteriales	Phormidiaceae	*Lyngbya_PCC-7,419*	MDM
0.831	Bacteria	Cyanobacteria	Cyanobacteria	Cyanobacteriales	MDM	MDM	MDM
0.816	MDM	MDM	MDM	MDM	MDM	MDM	MDM
0.810	MDM	MDM	MDM	MDM	MDM	MDM	MDM
0.807	Bacteria	Cyanobacteria	Cyanobacteria	Cyanobacteriales	Xenococcaceae	MDM	MDM
0.803	MDM	MDM	MDM	MDM	MDM	MDM	MDM
0.800	Bacteria	Cyanobacteria	Cyanobacteria	Cyanobacteriales	Xenococcaceae	MDM	MDM
0.797	MDM	MDM	MDM	MDM	MDM	MDM	MDM
0.795	MDM	MDM	MDM	MDM	MDM	MDM	MDM
0.794	MDM	MDM	MDM	MDM	MDM	MDM	MDM
0.794	Bacteria	Cyanobacteria	Cyanobacteria	MDM	MDM	MDM	MDM
0.789	Bacteria	Cyanobacteria	Cyanobacteria	Cyanobacteriales	MDM	MDM	MDM
0.786	Bacteria	Cyanobacteria	Cyanobacteria	MDM	MDM	MDM	MDM
0.781	Bacteria	Cyanobacteria	Cyanobacteria	Cyanobacteriales	Microcystaceae	MDM	MDM
0.773	Bacteria	Planctomycetota	Phycisphaerae	Phycisphaerales	Phycisphaeraceae	*SM1A02*	MDM
0.770	MDM	MDM	MDM	MDM	MDM	MDM	MDM

MDM, microbial dark matter, or unknown taxa.

## 4. Discussion

In this study, we applied a network approach to model microbial interactions in microbialite-forming communities utilizing data from amplicon, metagenomic and metatranscriptomic data sets to understand the role of unknown taxa within these communities and extend the network methodology of MDMnets to other types of data. We analyzed how these different sequencing approaches impacted the detection of the unknown components in the microbialite-forming communities. The results of this study suggest that: (1) microbial dark matter is abundant within microbialite-forming communities and they play an important role in maintaining community structure; (2) unknown taxa occupy keystone positions within the microbialite community; and (3) different types of sequencing data can used for network analysis and provide different perspectives and insight into microbial communities.

Amplicon sequencing is often the preferred method to investigate the composition of a microbial ecosystem and studies have shown that utilizing amplicon data to model microbial communities using co-occurrence networks can lead to the identification of unknown keystone taxa (Zamkovaya et al., 2021). Network approaches can lead to a better understanding of which organisms to prioritize for subsequent sequencing and characterization. In this study, we adapted the previously described methodology to other types of sequencing data to understand to which extent the type of sequencing profiling biases the assessment of unknown taxa relevance. Our strategy for adaptation of the MDMnets approach to metagenomic and metatranscriptomic data was to extract SSU reads from these datasets to subsequently process in a similar way as amplicon data. An expansion of this approach to include both metagenomic and metatranscriptomic data can provide valuable insight into active members of the community. Furthermore, the adaptation of this methodology to metagenomics and metatranscriptomics data allows the mining of these data in cases where no other data type is available. Analysis of the networks generated for all three datasets showed that uncultured and unsequenced microbes were highly abundant in microbialite-forming communities, regardless of origin, type and method used.

To make sure the obtained SSU sequences from metagenomics and metatranscriptomics had enough information for taxonomic classification, we applied a filter based on percentage of identity to the sequences on the SILVA database, which resulted in a significant reduction of the number sequences considered for network analysis. While this filtering approach guarantees the removal of low-quality sequences from our analysis, it may also exclude unknown taxa with divergent SSU genes present in our ecosystem, reflecting a limitation of current databases. Still, many these high information content sequences could not be taxonomically assigned at levels such as phylum, class or order, reflecting the widespread prevalence of insufficiently characterized microorganisms within the microbialite environments.

Our study found that the exclusion of unknown taxa from the network caused an alteration in network metrics for all datasets, showing that “microbial dark matter” was not only abundant in microbialites, but they also comprised an important component of the metabolically active fraction.

Typically, different network metrics normally indicate various characteristics of the microbial environment (Proulx et al., 2005). Degree centrality represents nodes that interact with most members in the network, whereas betweenness centrality refers to those nodes that have a high influence on the flow of information in a graph and closeness centrality describes nodes that are central to a network. Analyzing these metrics provided insight into the importance of different nodes within the community. For example, nodes that exhibited both high degree and high betweenness centrality were identified as hubs and may represent the most important members within the microbialite community as they were involved in many interactions and connections. Thus, their removal altered the structure of the network and resulted in the fragmentation.

A deeper look into each individual data type showed differences in taxonomic identification and unknown taxa prevalence between amplicon, metagenomic and metatranscriptomic. However, a core of 10 phyla were identified by all three methods (i.e., Proteobacteria, Bacteroidota, Myxococcota, Cyanobacteria, Planctomycetes, Chloroflexi, Desulfobacterota, NB1-j, Acidobacteriota and Bdellovibrionota) out of 23 different identified phyla classifications, revealing the presence a consistent microbial identity for the environment regardless the genomics assay applied, and the utilization of public data obtained from different sources and locations. Observed differences across datasets may reflect the sampling and sequencing strategy used in each case. Amplicon sequencing consists of the targeted sequencing of hypervariable regions within the highly conserved 16S rRNA marker gene. These regions are then utilized for taxonomy profiling, however, the adequate classification of taxa through amplicon sequencing is affected by a variety of biases, such as the choice of hypervariable region to utilize (Chakravorty et al., 2007; Haas et al., 2011) or even PCR or sequencing errors generated during library preparation. These biases impact both community profiling and diversity estimation in datasets (Smith et al., 2012; Nelson et al., 2014; Salipante et al., 2014; Brooks et al., 2015; Onywera and Meiring, 2020).

Unlike the amplicon dataset, metagenomic sequencing is a shotgun approach that aims to sample the collective genome of the microbial ecosystem in any chosen environment. Previous studies have shown that when comparing 16S rRNA gene sequencing with whole metagenome, the resulting microbial profiles are often different. Specifically, shotgun sequencing methods have a difficulty capturing lowly abundant species (Zhang et al., 2021; Jin et al., 2022) or lowly expressed genes in metatranscriptomic datasets (Kuske et al., 2015).

Our results indicate that the lack of amplification bias in metagenomic and metatranscriptomic sequencing could be the reason for the higher abundance of unknown taxa in these datasets when compared to amplicon data. Although amplicon sequencing introduces biases by including an amplification step that may exclude taxa that cannot be amplified with the normally used primers, this approach can help reduce the number of queried sequences, thus better capturing lowly-expressed or lowly-abundant sequences.

Despite having a similar approach and biases, metagenomic and metatranscriptomic approaches represent different aspects of a microbial community. While the metagenomic networks reflected the abundance of unknown taxa in microbialite-forming communities without the amplification biases of amplicon sequencing, metatranscriptomic networks revealed their high transcriptional activity. In addition, the extraction of SSU reads from metatranscriptomics results in the identification of more taxa than in metagenomics, as SSU genes are highly expressed, whereas in metagenomics the SSU genes represent a small portion of the genome, which results in less identified taxa. Here, we were able to show that the network approach can be used with different types of meta-omics data. Additionally, the adaptation of this methodology to different types of data revealed unknown keystone taxa that are abundant and occupy active metabolic roles within the microbialite-forming communities. This insight can be used to prioritize taxa for downstream characterization. Finally, our results show that the capacity for detecting “microbial dark matter” depends on the adopted-omics technology, and their different strengths and biases must be considered.

## 5. Conclusion

“Microbial dark matter” is highly prevalent and active in microbialite-forming communities. Using different sequencing methodologies, we were able to apply a co-occurrence network approach to understand community structure and find keystone taxa. Furthermore, the use of different methodologies allowed us to measure different aspects of the biological system, such as taxa presence or absence and the transcriptionally active portion. However, methods present different biases that may impact analysis results. While our study illustrates the gap of knowledge in the composition of microbialite-forming communities, this work provides a methodology to identify and prioritize taxa for downstream analyses.

## Data availability statement

The original contributions presented in the study are included in the article/Supplementary material, further inquiries can be directed to the corresponding authors.

## Author contributions

RA analyzed the data and wrote the original manuscript. All authors contributed to the article and approved the submitted version.

## Funding

Funding for this project was provided by the NASA Astrobiology Exobiology Program (80NSSC20K0612) awarded to AC and JF.

## Conflict of interest

The authors declare that the research was conducted in the absence of any commercial or financial relationships that could be construed as a potential conflict of interest.

## Publisher’s note

All claims expressed in this article are solely those of the authors and do not necessarily represent those of their affiliated organizations, or those of the publisher, the editors and the reviewers. Any product that may be evaluated in this article, or claim that may be made by its manufacturer, is not guaranteed or endorsed by the publisher.
