# Supplementary material for: Integration of multi-omics data to elucidate keystone unknown taxa within microbialite-forming ecosystems
Source: Front Microbiol. 2023 Jul 28;14:1174685. doi: 10.3389/fmicb.2023.1174685 (PMC10416242; doi:10.3389/fmicb.2023.1174685)
Supplement: Supplementary file 2 [file Data_Sheet_1.PDF]

Supplementary Material

Integration of multi-omics data to elucidate keystone unknown taxa within microbialite-forming ecosystems

Rocío Amorín de Hegedüs[1,3], Ana Conesa[2*], Jamie S. Foster[3*]

[1]Genetics Institute, University of Florida, Gainesville, FL, USA
[2]Spanish National Research Council, Institute for Integrative Systems Biology, València, Spain
[3]Department of Microbiology and Cell Sciences, Space Life Sciences Lab, University of Florida, Merritt Island, FL, USA

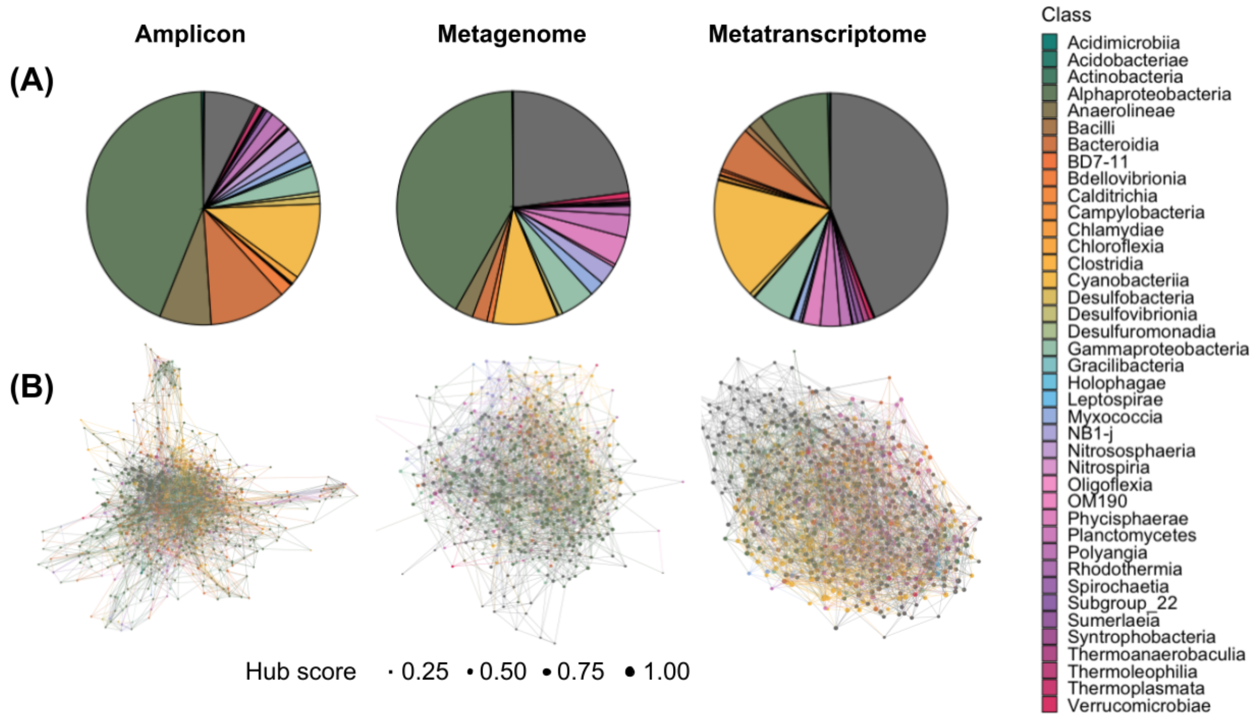

Supplementary Figure 1. Overall diversity of the datasets at the class taxonomic level. **(A)** Distribution of taxa abundance in each dataset demonstrating a high proportion of 'microbial dark matter' present in the metagenomic and metatranscriptomic datasets. **(B)** Hub network at the class level, hub size indicates hub score, with one being highest hub score and zero being lowest. Each network represents the three types of data depicting the distinctive patterns of unknown organisms, labeled as 'microbial dark matter', within the microbialite communities.

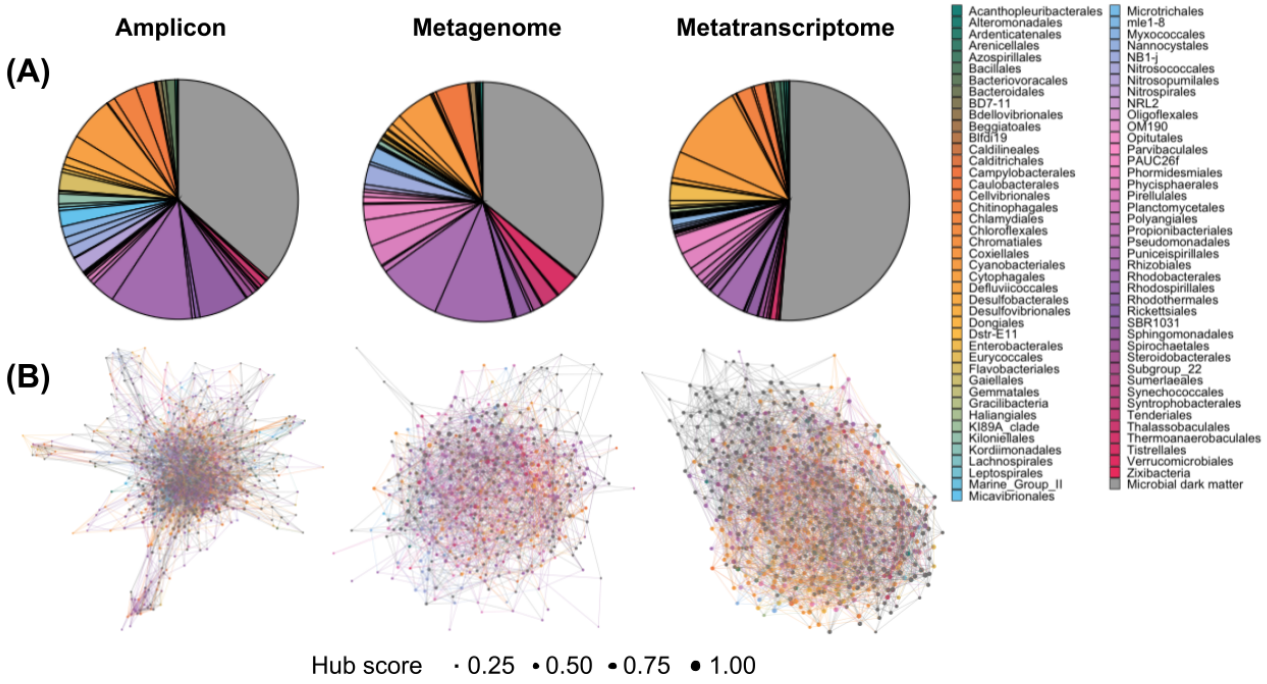

Supplementary Figure 2. Overall diversity of the datasets at the order taxonomic level. **(A)** Distribution of taxa abundance in each dataset demonstrating a high proportion of microbial dark matter present in the metagenomic and metatranscriptomic datasets. **(B)** Hub network at the order level, hub size indicates hub score, with one being highest hub score and zero being lowest. Each network represents the three types of data depicting the distinctive patterns of unknown organisms, labeled as 'microbial dark matter', within the microbialite communities.

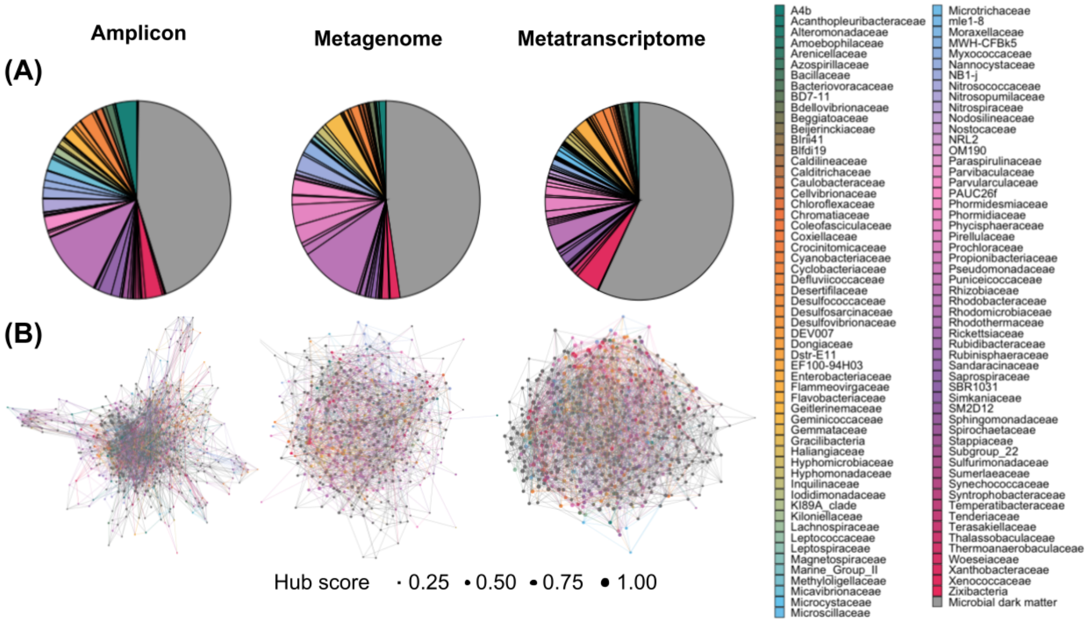

Supplementary Figure 3. Overall diversity of the datasets at the family taxonomic level. **(A)** Distribution of taxa abundance in each dataset demonstrating a high proportion of microbial dark matter present in the metagenomic and metatranscriptomic datasets. **(B)** Hub network at the family level, hub size indicates hub score, with one being highest hub score and zero being lowest. Each network represents the three types of data depicting the distinctive patterns of unknown organisms, labeled as 'microbial dark matter', within the microbialite communities.

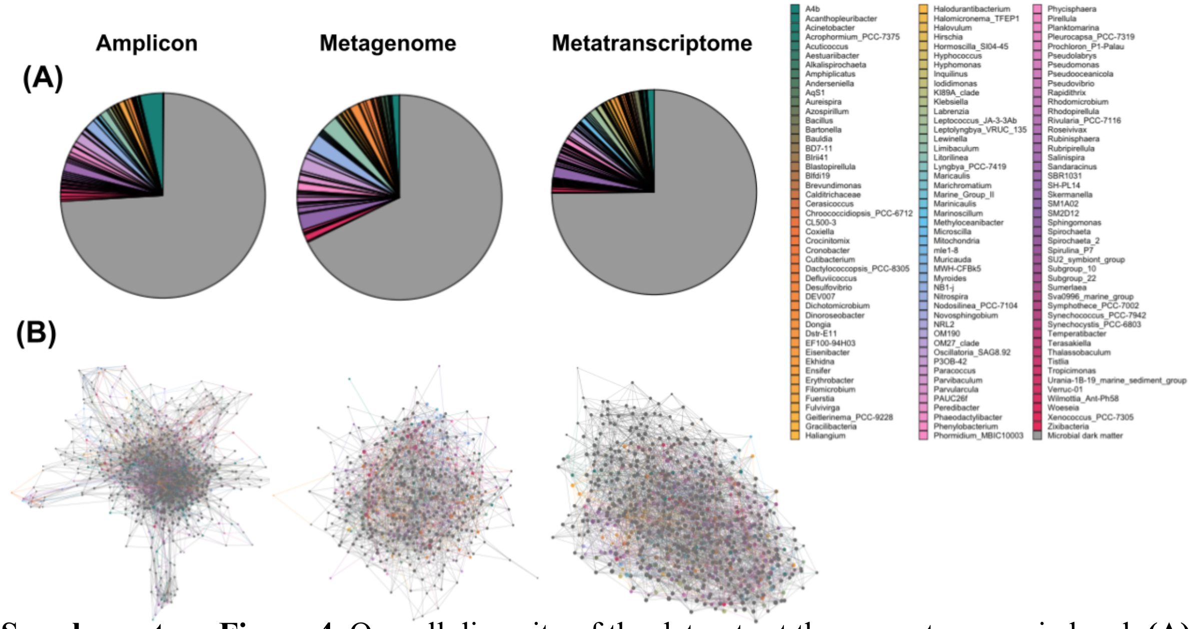

Supplementary Figure 4. Overall diversity of the datasets at the genus taxonomic level. **(A)** Distribution of taxa abundance in each dataset demonstrating a high proportion of microbial dark matter present in the metagenomic and metatranscriptomic datasets. **(B)** Hub network at the genus level, hub size indicates hub score, with one being highest hub score and zero being lowest. Each network represents the three types of data depicting the distinctive patterns of unknown organisms, labeled as 'microbial dark matter', within the microbialite communities.

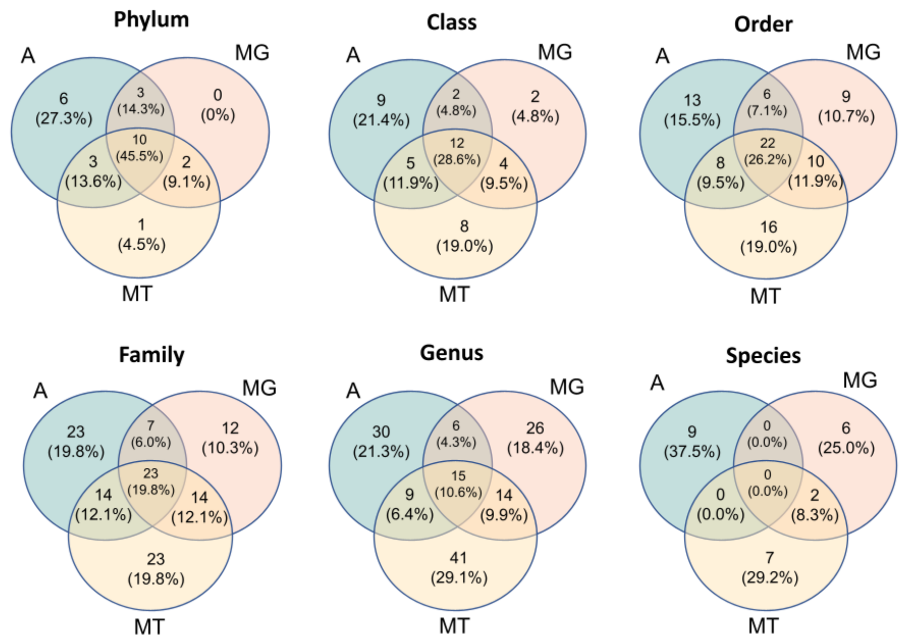

Supplementary Figure 5. Venn diagram of microbial dark matter shared amongst the amplicon (A), metagenome (MG) and metatranscriptome (MT) datasets at all taxonomic levels.

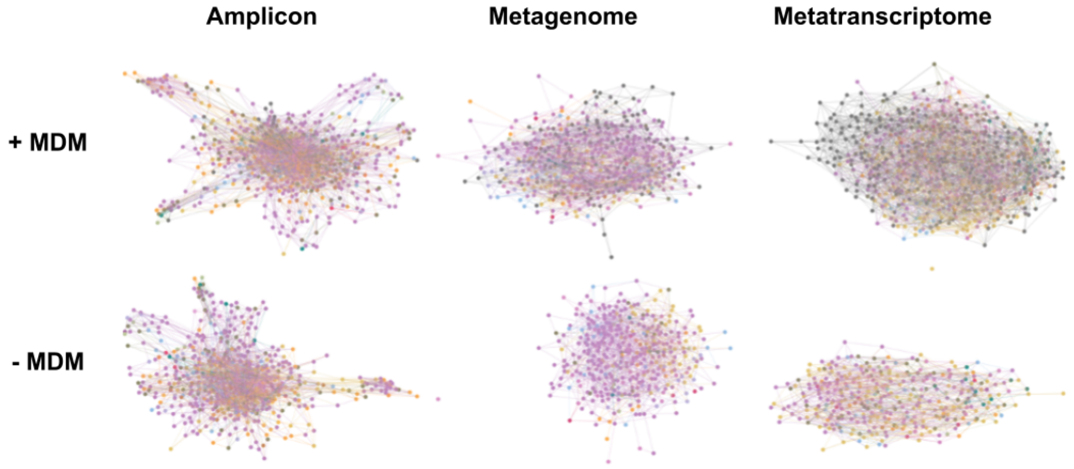

Supplementary Figure 6. Networks created at the phyla level for amplicon, metagenome and metatranscriptome datasets showing structural changes in network connectivity when comparing networks created with and without microbial dark matter (MDM).

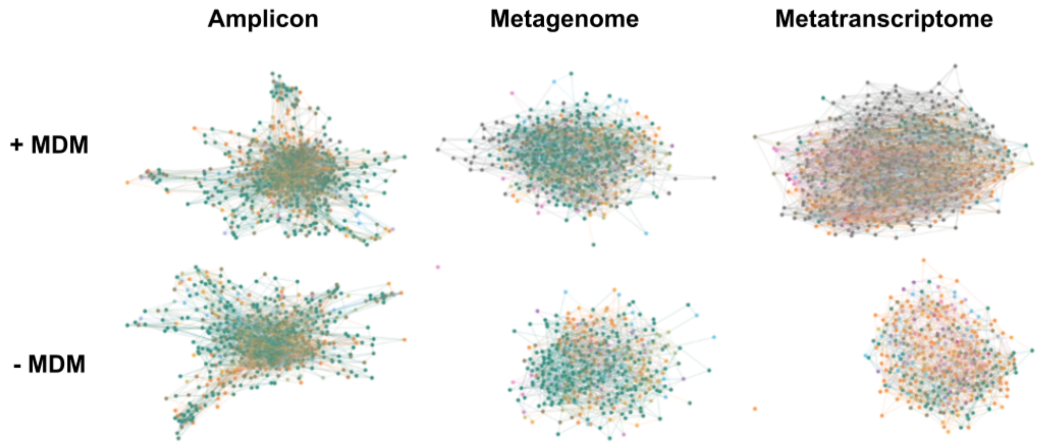

Supplementary Figure 7. Networks created at the class level for amplicon, metagenome and metatranscriptome datasets showing structural changes in network connectivity when comparing networks created with and without microbial dark matter (MDM).

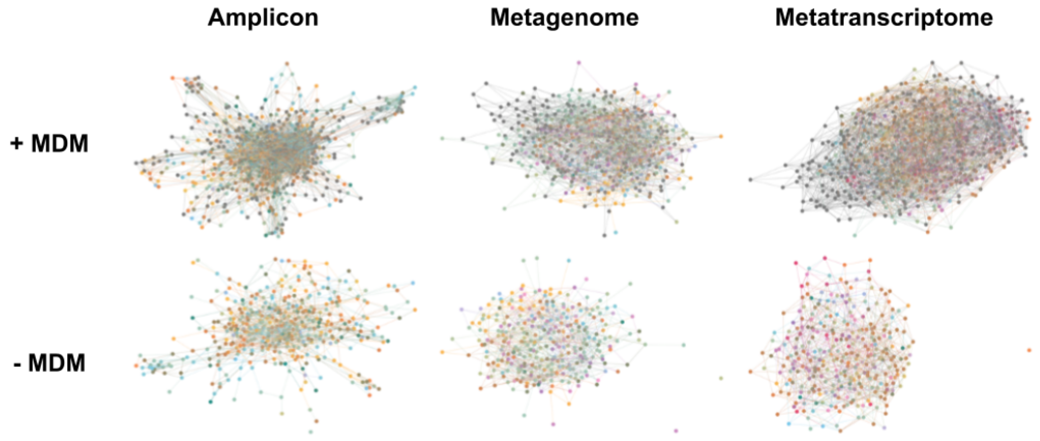

Supplementary Figure 8. Networks created at the order level for amplicon, metagenome and metatranscriptome datasets showing structural changes in network connectivity when comparing networks created with and without microbial dark matter (MDM).

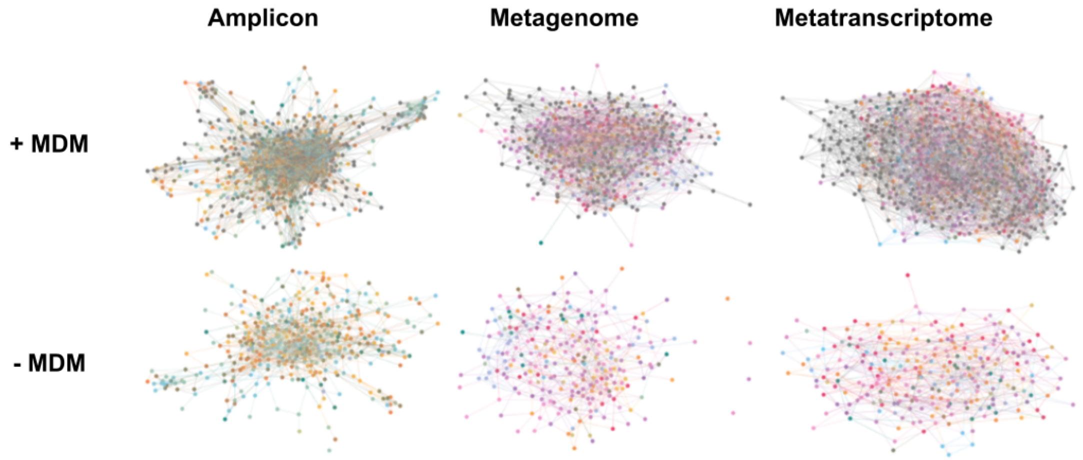

Supplementary Figure 9. Networks created at the family level for amplicon, metagenome and metatranscriptome datasets showing structural changes in network connectivity when comparing networks created with and without microbial dark matter (MDM).

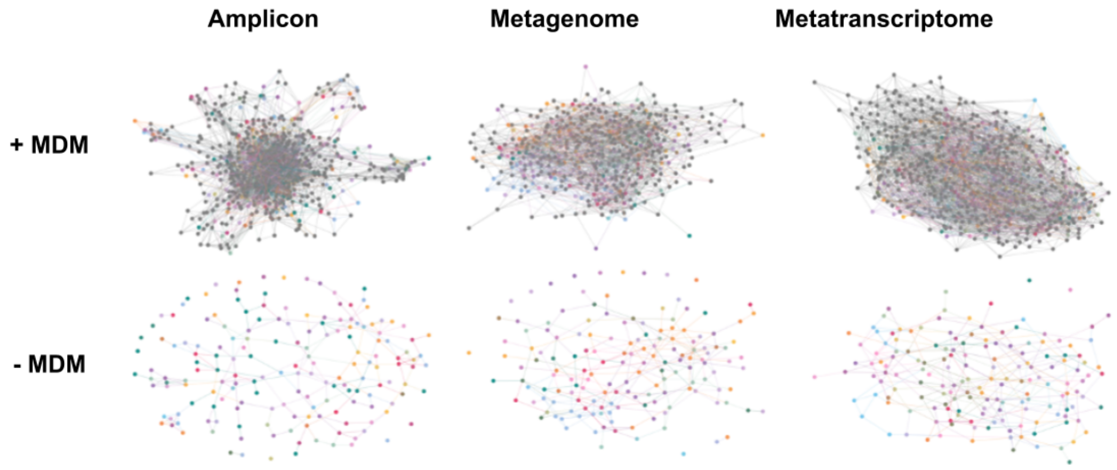

Supplementary Figure 10. Networks created at the genus level for amplicon, metagenome and metatranscriptome datasets showing structural changes in network connectivity when comparing networks created with and without microbial dark matter (MDM).

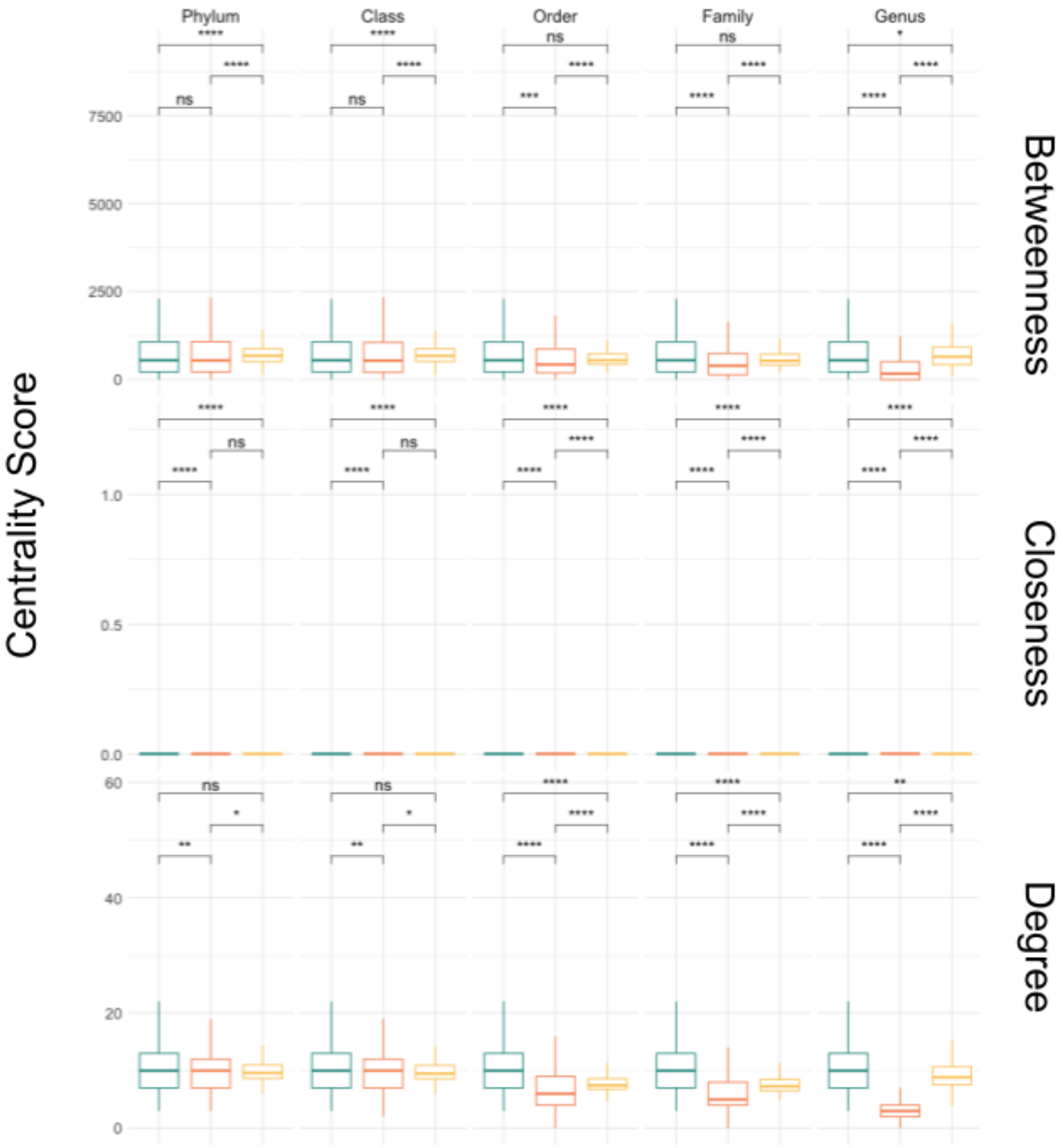

Supplementary Figure 11. Comparison of centrality scores for the amplicon dataset between the original network (green), the network without unknown taxa (orange) and the bootstrap network at all taxonomic levels (yellow). The evaluated network metrics of the hubs represent connectivity including betweenness centrality, closeness centrality and co-occurrence (i.e., degree centrality) (ns, $p > 0.05$; *, $p \leq 0.05$; ** $p \leq 0.01$; ***, $p \leq 0.001$; ****, $p \leq 0.0001$).

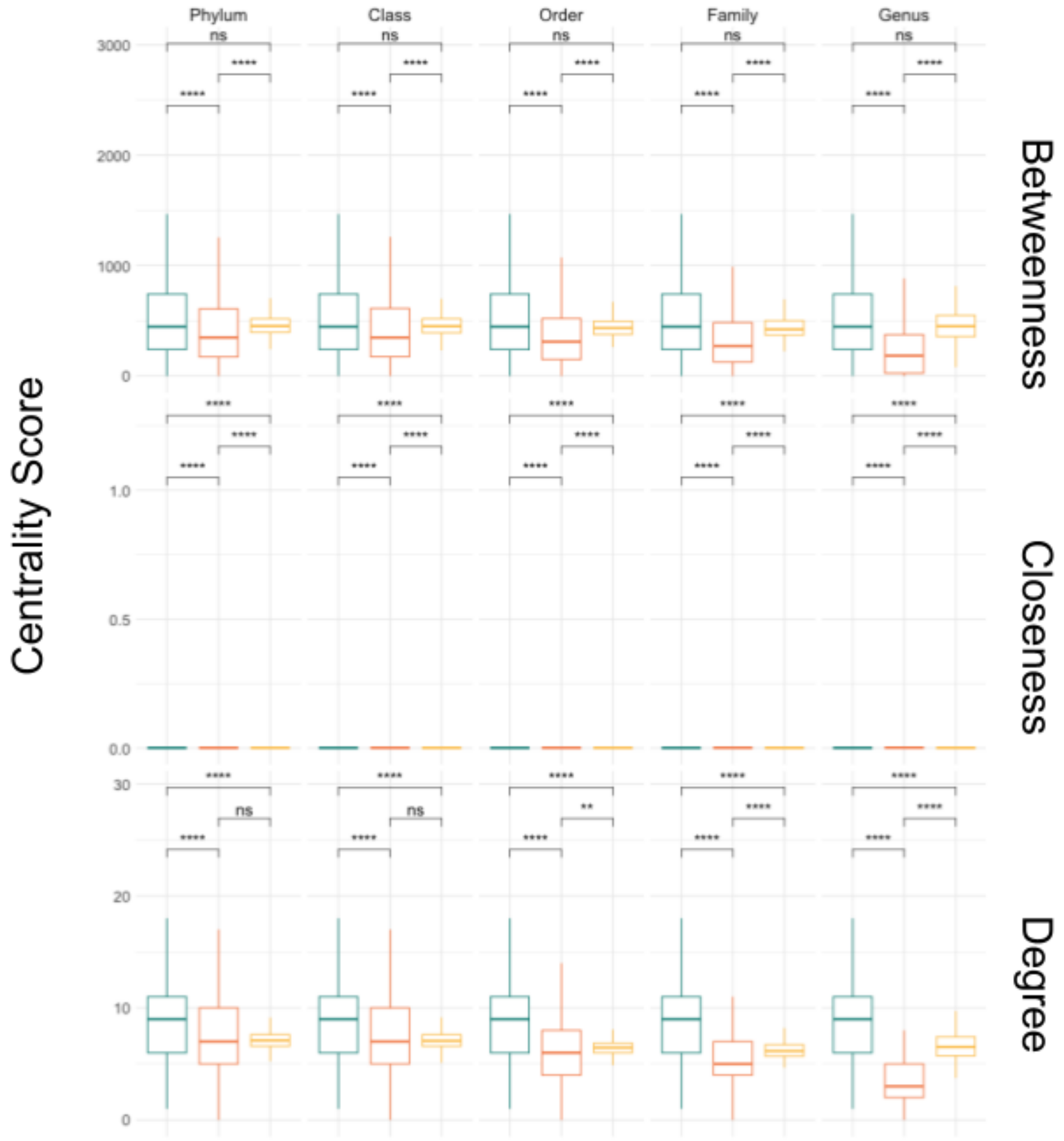

Supplementary Figure 12. Comparison of centrality scores for the metagenomic dataset between the original network (green), the network without unknown taxa (orange) and the bootstrap network at all taxonomic levels (yellow). The evaluated network metrics of the hubs represent connectivity including betweenness centrality, closeness centrality and co-occurrence (i.e., degree centrality) (ns, $p > 0.05$; *, $p \leq 0.05$; ** $p \leq 0.01$; ***, $p \leq 0.001$; ****, $p \leq 0.0001$).

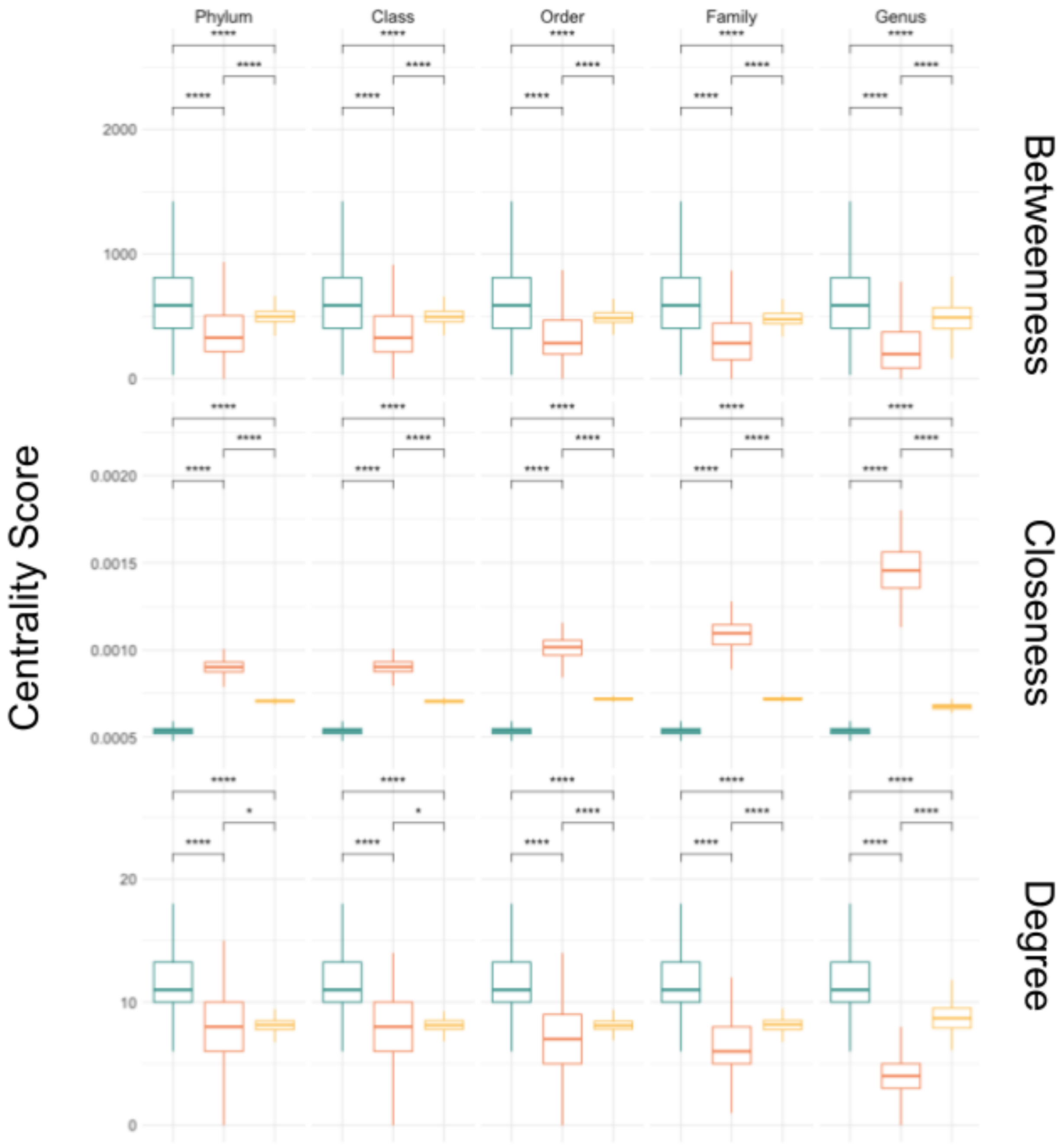

Supplementary Figure 13. Comparison of centrality scores for the metatranscriptomic dataset between the original network (green), the network without unknown taxa (orange) and the bootstrap network at all taxonomic levels (yellow). The evaluated network metrics of the hubs represent connectivity including betweenness centrality, closeness centrality and co-occurrence (i.e., degree centrality) (ns, $p > 0.05$; *, $p \leq 0.05$; ** $p \leq 0.01$; ***, $p \leq 0.001$; ****, $p \leq 0.0001$).

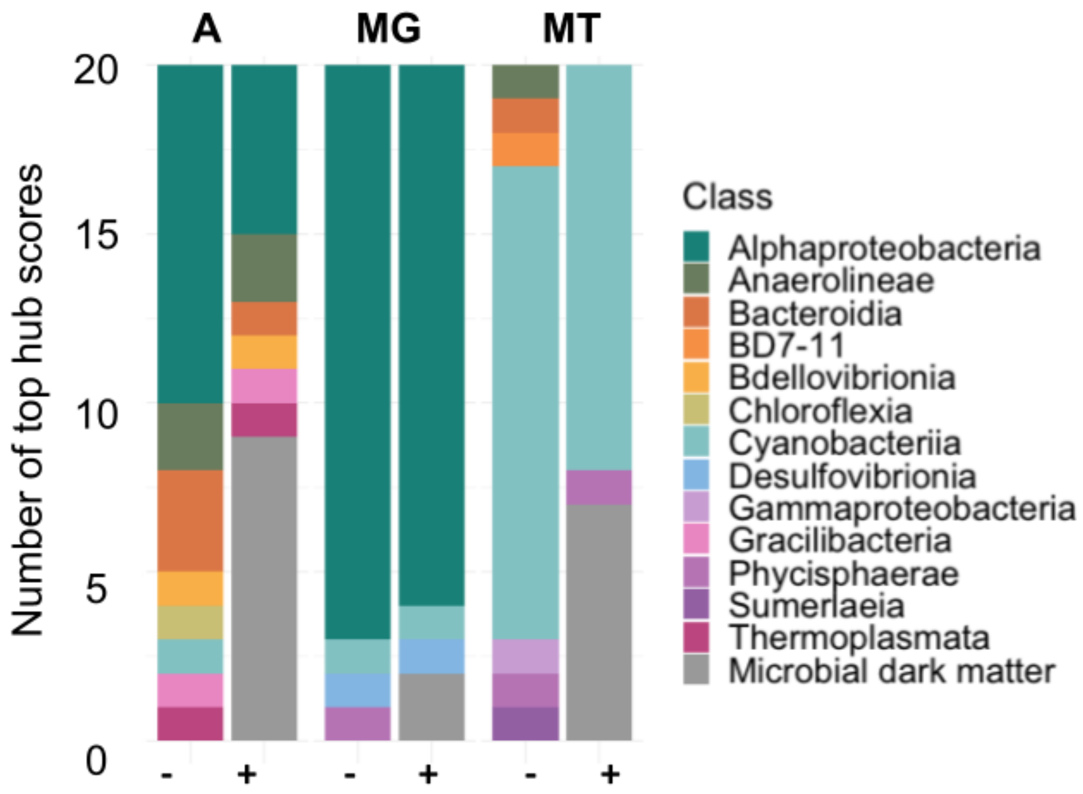

Supplementary Figure 14. Bar plots indicating microbial diversity at the class level for top 20 hubs in the presence (+) and absence (-) of microbial dark matter in the amplicon (A), metagenome (MG) and metatranscriptome (MT) datasets.

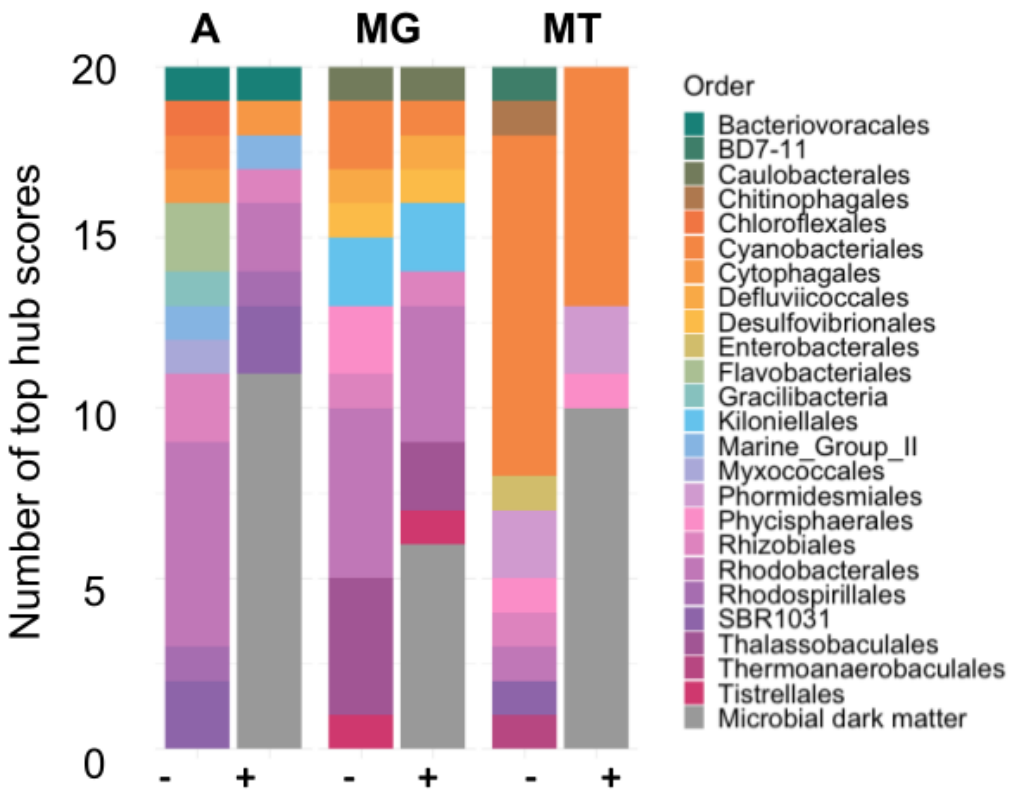

Supplementary Figure 15. Bar plots indicating microbial diversity at the order level for top 20 hubs in the presence (+) and absence (-) of microbial dark matter in the amplicon (A), metagenome (MG) and metatranscriptome (MT) datasets.

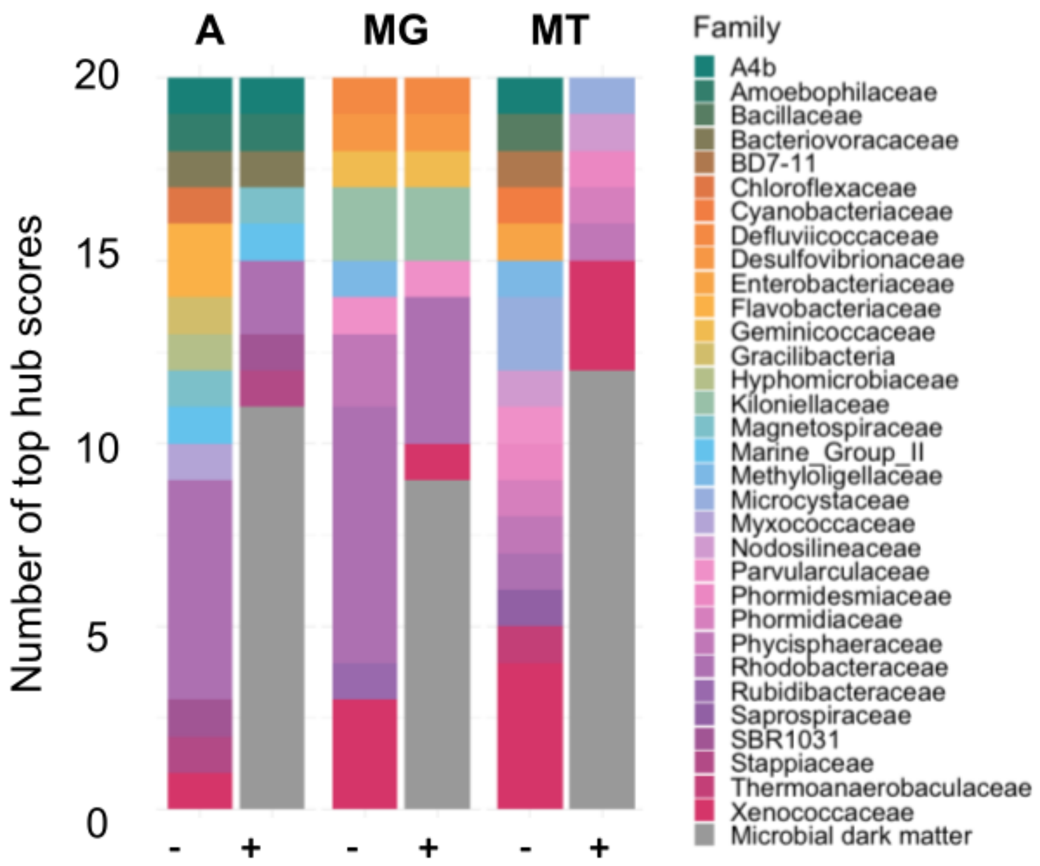

Supplementary Figure 16. Bar plots indicating microbial diversity at the family level for top 20 hubs in the presence (+) and absence (-) of microbial dark matter in the amplicon (A), metagenome (MG) and metatranscriptome (MT) datasets.

Supplemental Table 1. Data used in the current study and recovered 16S rRNA genes extracted from data

Sample Type	Sample Name*	Before Trimming	After Trimming	Percentage filtered	Extracted by SortMeRNA	Percentage extracted	Publication Source of Data
Amplicon	Top_0_3mm_1_AB	17,014	16,712	1.78	n/a	n/a	Mobberley et al., 2015
Amplicon	Top_0_3mm_2_AB	12,665	12,533	1.04	n/a	n/a	Mobberley et al., 2015
Amplicon	Top_0_3mm_3_AB	16,992	16,797	1.15	n/a	n/a	Mobberley et al., 2015
Amplicon	Middle_3_5mm_1_AB	20,065	19,855	1.05	n/a	n/a	Mobberley et al., 2015
Amplicon	Middle_3_5mm_2_AB	13,409	13,289	0.89	n/a	n/a	Mobberley et al., 2015
Amplicon	Middle_3_5mm_3_AB	17,807	17,627	1.01	n/a	n/a	Mobberley et al., 2015
Amplicon	Bottom_5_9mm_1_AB	20,566	20,341	1.09	n/a	n/a	Mobberley et al., 2015
Amplicon	Bottom_5_9mm_2_AB	23,052	22,839	0.92	n/a	n/a	Mobberley et al., 2015
Amplicon	Bottom_5_9mm_3_AB	19,142	18,873	1.41	n/a	n/a	Mobberley et al., 2015
Amplicon	Calcareous_Knobs	35,854	34,277	4.4	n/a	n/a	Paul et al., 2016
Amplicon	Cheesecake_Mat	35,718	32,219	9.8	n/a	n/a	Paul et al., 2016
Amplicon	Mushroom_Mat	26,296	22,883	12.98	n/a	n/a	Paul et al., 2016
Amplicon	Pinnacle_Mound	23,082	18,603	19.4	n/a	n/a	Paul et al., 2016
Amplicon	Plateau_Mats	32,512	31,848	2.04	n/a	n/a	Paul et al., 2016
Metagenome	bockcaya	37,332,915	34,610,103	7.29	718,842	2.08	Casaburi et al., 2017
Metagenome	bockcayb	14,780,796	12,998,008	12.06	196,135	1.51	Casaburi et al., 2017
Metagenome	bockcayc	14,256,446	12,654,877	11.23	193,113	1.53	Casaburi et al., 2017
Metagenome	darbya	46,544,696	43,340,598	6.88	597,646	1.38	Casaburi et al., 2017
Metagenome	darbyb	17,597,932	15,903,579	9.63	243,051	1.53	Casaburi et al., 2017
Metagenome	darbyc	15,714,314	14,263,136	9.23	190,198	1.33	Casaburi et al., 2017
Metagenome	gga	28,571,849	25,950,654	9.17	340,942	1.31	Casaburi et al., 2017
Metagenome	ggb	27,038,086	24,577,038	9.1	279,166	1.14	Casaburi et al., 2017
Metagenome	ggc	26,928,030	24,437,635	9.25	313,986	1.28	Casaburi et al., 2017
Metagenome	lsia	37,873,705	35,530,613	6.19	312,971	0.88	Casaburi et al., 2017
Metagenome	lsib	15,318,518	14,020,710	8.47	144,111	1.03	Casaburi et al., 2017
Metagenome	lsic	17,016,636	15,446,260	9.23	171,942	1.11	Casaburi et al., 2017
Metagenome	m11a	40,342,944	37,650,506	6.67	298,545	0.79	Babilonia et al., 2018
Metagenome	m11b	15,537,932	13,593,996	12.51	158,610	1.17	Babilonia et al., 2018
Metagenome	m11c	14,902,519	13,188,242	11.5	118,075	0.9	Babilonia et al., 2018
Metagenome	m13a	35,835,582	33,373,392	6.87	311,355	0.93	Babilonia et al., 2018
Metagenome	m13b	16,894,618	15,025,727	11.06	78,801	0.52	Babilonia et al., 2018
Metagenome	m13c	13,295,840	12,187,295	8.34	47,384	0.39	Babilonia et al., 2018
Metagenome	m30	25,405,384	23,136,332	8.93	323,919	1.4	Babilonia et al., 2018
Metagenome	m33	24,771,072	22,443,684	9.4	341,209	1.52	Babilonia et al., 2018
Metagenome	m34	31,005,027	27,940,412	9.88	439,223	1.57	Babilonia et al., 2018
Metagenome	m40a	12,417,269	10,725,012	13.63	136,851	1.28	Babilonia et al., 2018
Metagenome	m40b	15,328,531	13,831,027	9.77	97,372	0.7	Babilonia et al., 2018
Metagenome	m40c	24,359,663	21,974,618	9.79	317,706	1.45	Babilonia et al., 2018
Metagenome	m41	25,960,540	23,596,004	9.11	333,457	1.41	Babilonia et al., 2018
Metagenome	m42	39,234,154	36,643,661	6.6	386,325	1.05	Babilonia et al., 2018
Metagenome	m47a	45,651,183	42,728,921	6.4	462,544	1.08	Babilonia et al., 2018
Metagenome	m47c	15,131,871	13,606,290	10.08	98,943	0.73	Babilonia et al., 2018
Metagenome	m48	26,523,578	24,071,331	9.25	275,433	1.14	Babilonia et al., 2018
Metagenome	m51a	25,651,724	23,000,237	10.34	282,834	1.23	Babilonia et al., 2018
Metagenome	m51b	26,269,911	23,627,178	10.06	299,935	1.27	Babilonia et al., 2018
Metagenome	m51c	26,761,266	23,734,078	11.31	331,562	1.4	Babilonia et al., 2018
Metagenome	m57	27,804,352	25,227,617	9.27	277,932	1.1	Babilonia et al., 2018
Metagenome	m5a	41,732,821	39,413,787	5.56	341,840	0.87	Babilonia et al., 2018
Metagenome	m5b	14,283,743	12,643,823	11.48	125,925	1	Babilonia et al., 2018
Metagenome	m5c	15,768,550	13,717,478	13.01	141,275	1.03	Babilonia et al., 2018
Metagenome	m66	30,425,854	27,228,944	10.51	274,268	1.01	Babilonia et al., 2018
Metagenome	m67	24,934,207	22,141,179	11.2	306,027	1.38	Babilonia et al., 2018
Metagenome	m74	26,175,923	23,859,400	8.85	309,104	1.3	Babilonia et al., 2018
Metagenome	m80a	33,336,796	31,241,413	6.29	282,504	0.9	Babilonia et al., 2018
Metagenome	m80b	14,282,307	12,341,336	13.59	205,780	1.67	Babilonia et al., 2018
Metagenome	m80c	16,857,531	15,096,040	10.45	176,876	1.17	Babilonia et al., 2018
Metagenome	m84	26,518,635	23,922,289	9.79	291,818	1.22	Babilonia et al., 2018
Metagenome	m86	25,244,020	22,789,400	9.72	267,623	1.17	Babilonia et al., 2018
Metagenome	m90	24,914,923	22,565,593	9.43	328,984	1.46	Babilonia et al., 2018
Metagenome	m93	23,846,052	21,621,270	9.33	244,480	1.13	Babilonia et al., 2018
Metagenome	t1a	35,119,170	32,333,541	7.93	372,340	1.15	Babilonia et al., 2018
Metagenome	t1b	14,927,579	13,571,138	9.09	152,194	1.12	Babilonia et al., 2018
Metagenome	t1c	17,094,507	15,632,953	8.55	178,059	1.14	Babilonia et al., 2018
Metagenome	t2a	45,533,186	42,604,335	6.43	416,136	0.98	Babilonia et al., 2018
Metagenome	t2b	17,372,395	15,874,359	8.62	175,689	1.11	Babilonia et al., 2018
Metagenome	t2c	15,880,346	14,569,859	8.25	155,090	1.06	Babilonia et al., 2018
Metagenome	t3a	42,795,349	39,879,708	6.81	525,778	1.32	Babilonia et al., 2018
Metagenome	t3b	15,367,706	13,923,348	9.4	145,984	1.05	Babilonia et al., 2018
Metagenome	t3c	16,437,667	15,100,449	8.14	168,688	1.12	Babilonia et al., 2018

Supplemental Table 1. Continued

Sample Type	Sample Name*	Before Trimming	After Trimming	Percentage filtered	Extracted by SortMeRNA	Percentage extracted	Publication Source of Data
Metatranscriptome	ad1t12	15,683,546	14,921,394.00	4.86	12,590,261	84.38	Louyakis et al., 2018
Metatranscriptome	ad1t18	31,787,779	30,191,725.00	5.02	20,107,306	66.6	Louyakis et al., 2018
Metatranscriptome	ad1t24	23,199,244	22,079,680.00	4.83	15,183,557	68.77	Louyakis et al., 2018
Metatranscriptome	ad1t6	19,485,124	18,639,717.00	4.34	13,203,813	70.84	Louyakis et al., 2018
Metatranscriptome	ad3t12	27,577,149	26,230,835.00	4.88	18,882,591	71.99	Louyakis et al., 2018
Metatranscriptome	ad3t18	27,545,235	26,167,411.00	5	19,965,126	76.3	Louyakis et al., 2018
Metatranscriptome	ad3t24	24,947,672	23,761,568.00	4.75	18,548,773	78.06	Louyakis et al., 2018
Metatranscriptome	ad3t6	25,896,494	24,387,079.00	5.83	17,444,005	71.53	Louyakis et al., 2018
Metatranscriptome	md2t12	21,981,478	20,695,443.00	5.85	10,917,770	52.75	Louyakis et al., 2018
Metatranscriptome	md2t18	9,020,386	8,460,090.00	6.21	4,633,300	54.77	Louyakis et al., 2018
Metatranscriptome	md2t24	15,179,281	14,049,061.00	7.45	6,922,153	49.27	Louyakis et al., 2018
Metatranscriptome	md2t6	21,143,468	20,088,733.00	4.99	15,808,657	78.69	Louyakis et al., 2018
Metatranscriptome	md3t12	17,404,602	16,406,706.00	5.73	11,874,923	72.38	Louyakis et al., 2018
Metatranscriptome	md3t18	23,602,509	22,237,407.00	5.78	9,627,174	43.29	Louyakis et al., 2018
Metatranscriptome	md3t24	39,423,204	37,154,880.00	5.75	20,041,889	53.94	Louyakis et al., 2018
Metatranscriptome	md3t6	25,398,177	23,965,744.00	5.64	13,096,101	54.65	Louyakis et al., 2018
Metatranscriptome	od2t12	38,972,757	36,875,439.00	5.38	23,596,741	63.99	Louyakis et al., 2018
Metatranscriptome	od2t18	35,716,740	34,076,695.00	4.59	21,872,141	64.19	Louyakis et al., 2018
Metatranscriptome	od2t24	19,689,798	18,664,620.00	5.21	13,973,042	74.86	Louyakis et al., 2018
Metatranscriptome	od2y6	19,804,547	18,856,430.00	4.79	13,925,093	73.85	Louyakis et al., 2018
Metatranscriptome	od3t12	37,855,938	35,863,276.00	5.26	19,891,046	55.46	Louyakis et al., 2018
Metatranscriptome	od3t18	23,425,928	22,117,737.00	5.58	15,706,506	71.01	Louyakis et al., 2018
Metatranscriptome	od3t24	37,892,570	36,100,307.00	4.73	27,229,236	75.43	Louyakis et al., 2018
Metatranscriptome	od3t6	17,522,040	16,485,760.00	5.91	11,604,562	70.39	Louyakis et al., 2018

*sample names derived from original publication of the data types
